# A matter of choice: a cross-sectional study examining the impact of the overturning of *Roe v Wade* on U.S. medical students’ perceptions and career decisions

**DOI:** 10.12688/mep.20519.1

**Published:** 2024-08-16

**Authors:** Alissa Conklin, Zeb Saeed, Sacha Sharp

**Affiliations:** 1 Department of Obstetrics and Gynecology, Indiana University School of Medicine, Indianapolis, Indiana, USA; 2 Department of Medicine, Indiana University School of Medicine, Indianapolis, Indiana, USA

**Keywords:** abortion, education, career choices

## Abstract

**Background:**

In June 2022, the *Dobbs* decision by the U.S. Supreme Court overturned federal abortion protections. In states with restrictive abortion laws such as Indiana, which also has the country’s largest medical school and the third worst maternal mortality rate, the impact of this ruling may be huge on the medical field. The purpose of this study was to analyze perceptions of medical students in Indiana in their third and fourth years of education after the *Dobbs* decision to assess if the state’s current abortion restrictions impact their career choice.

**Methods:**

Between December 2022 and March 2023, an anonymous survey was carried out at Indiana University School of Medicine, which included questions about personal beliefs on abortion and the current abortion laws in Indiana, as well as priorities when choosing residency training and practice locations.

**Results:**

Our survey found that four-fifths of medical students in Indiana disagreed with the *Dobbs* decision. While most students (71.4%) had not considered state abortion laws when selecting a medical school, since the Dobbs decision, 66.3% of third-year and 40.3% of fourth-year students indicated that they would take abortion laws into account when choosing a residency program. 47.5% of female students stated that they will be seeking residency in a state where abortion is legal and 55.3% of single students were more likely to leave Indiana to practice medicine.

**Conclusion:**

Our research suggests that physicians who are more liberal in their views on abortion may now be much less likely to practice in conservative states which will compound the healthcare outcomes secondary to the Dobbs decision. We emphasize the role that abortion laws have in shaping the landscape of healthcare workforce and the need for a more nuanced understanding of how societal structures impact women's reproductive decisions and career paths in medicine.

## Introduction

Abortion has always been a contentious healthcare issue in the United States. In 1973, the U.S. Supreme Court ruled abortion as a right protected by the constitution, striking down several anti-abortion laws ^1^. However, in June 2022, the U.S. Supreme Court reversed that decision in *Dobbs v Jackson Women’s Health Organization,* stating the Constitution does not confer a right to abortion, effectively returning the power to regulate any aspect of abortion not protected by federal law back to individual states ^2, 3^. This ruling is in contradiction with the 2014 American College of Obstetricians and Gynecologists (ACOG) recommendation that abortion education be included in the curricula of all medical schools ^4^. In addition, the Association of Professors of Gynecology and Obstetrics (APGO) went one step further and asserted, “Regardless of personal views about abortion, students should be knowledgeable about its public health importance, as well as techniques and patient safety implications” ^5^. These conflicting priorities between the federal law and leading medical associations are at the center of reproductive healthcare education for medical school trainees. 

It is imperative not only to acknowledge the discrepancy between changes in federal law and what is regarded as an essential component of all obstetrics and gynecology (OBGYN) clerkships—a mandatory clinical experience for all United States medical students—but also to recognize that women are the demographic group most significantly impacted by abortion laws. Presently, women are the largest group of matriculants into medical school with 53.8% of matriculating students in the 2022–2023 school year being women ^6^. Regarding matching into residency, 2022 match data show that of the 1,836 students who matched into OB/GYN residency training programs, 86.4% identify as females ^7^. In fact, OB/GYN as a specialty has the highest proportion of female applicants and matched students of all specialties in the country ^7^. Hence female medical students are doubly impacted by this ruling, both as females concerned with their own bodily autonomy and as medical students trying to learn about safe abortion practices and care in a post- *Dobbs* era.

It is estimated that 9,505 (41.9%) matriculating medical students will receive their medical education in the 24 states that have prohibited or severely restricted abortion since the *Dobbs* decision ^8^. One of those states, Indiana, has the largest medical school in the country. Indiana also has the third highest maternal mortality rate in the country ^9^. Evidence points to worsening maternal mortality rates among states with restrictive laws on abortion. One study utilizing the 2018-2020 state-level maternal mortality and morbidity from the Healthcare Cost and Utilization Project database found that per 100,000 births, the maternal mortality rate was more than double in states with restrictive laws on abortion compared to states with protective laws on abortion (4.0 vs 9.3) ^10^. Medical educators in these states are concerned that newly graduating medical students may not wish to get their residency training or to stay within a state that is restrictive towards abortion, such as Indiana ^11, 12^. This is of particular concern for the health of state residents, as it is essential that the states retain and attract the highest capable physicians to reverse adverse healthcare statistics.

While access to abortion training is critical to medical education, all medical students and physicians, as well as their partners, are also patients. Access to their own adequate healthcare must be considered in the decision-making paradigm of life choices, including where to train and where to practice medicine. Almost one in four (23.7%) women in the United States will have an abortion by age 45 ^13^. Medical training often occurs during prime reproductive years. A 2021 survey of over 3,000 physicians and medical students who desired children found that 1 in 6 medical students or their partners had had an abortion ^14^. While this survey showed less frequent abortion among medical trainees and physicians compared to the general population, it focused only on those who desired children and therefore likely underrepresents abortion rates in the medical community.

The purpose of this study was to analyze the perceptions of Indiana medical students in their third and fourth years of education after the *Dobbs* decision and to assess if the state’s current abortion restrictions impact their career choice, location for residency training, and where they ultimately desire to practice and live ^15^. In Indiana, conversations about the practice of abortion have made national news. In the case of Dr. Caitlin Bernard, the nation has witnessed how a physician can be scrutinized and reprimanded for doing their job, and how abortion law restrictions and political motivations can disrupt work and life ^16^. Given the national spotlight, it is imperative to determine how the *Dobbs* decision may impact career decisions for medical students in Indiana.

## Methods

Between December 2022 and March 2023, a cross-sectional survey was carried out among third- and fourth-year medical students at Indiana University School of Medicine (See Extended data section below). The survey was anonymous and included questions about personal beliefs on abortion and the current abortion laws in Indiana, as well as priorities when choosing residency training and practice locations. Specifically, the survey asked about the influence of abortion laws on the students’ decision-making and whether students intended to stay in Indiana, considering the recent *Dobbs* decision. Basic demographic information was also collected. The survey was distributed twice via a student newsletter and once via direct email to all third- and fourth-year medical students. The study was granted exempt status and approved by the Indiana University IRB, IRB Number: 17160. SPSS 28.0 was used to analyze the data, with descriptive statistics performed. Chi-square was utilized to examine the associations between various demographic factors and participant responses. A p-value of <0.05 was considered statistically significant.

Concerning the research team, all members identify as cis-gender women. One of the research team members identifies as white, one identifies as Southeast Asian, and the final research member identifies as Black. Each team member serves as faculty at an academic medicine institution, and two of the three researchers work as clinicians in large medical centers. Their fields of specialty include obstetrics and gynecology and internal medicine. The research team members share a passion and responsibility for educating and treating individuals from underrepresented backgrounds.

We used structural competency to analyze the data and address our research question concerning medical students’ perceptions of the *Dobbs* decision and how it affects their career decisions. Within the competency framework, structures are defined as “policies, economic systems, and other institutions that have been produced and maintain social inequities and health disparities, often along the lines of social categories such as race, class, gender, and sexuality ^17^. Understanding how structures built within our society affect the health outcomes of our patients is an important aspect of making the necessary educational choices for training future physicians. Therefore, we particularly focused on rearticulating cultural presentations in structural terms and observing and imagining structural intervention as structural components relevant to our analysis when examining the data, with particular focus on women's experiences, agency and decision-making processes.

## Results

A total of 763 third- and fourth-year IU medical students were invited to participate in the study. Of them, 178 students initiated the survey, resulting in a response rate of 23.3%. Out of those who started the survey, 168 students completed it and were included in the final analysis. The demographic characteristics of the participants are summarized in Table 1 and are indicative of these classes’ heterogeneity. Our cohort consisted of 53.6% (89) females, primarily individuals aged 30 or younger (96.4%,158), identifying as heterosexual (76.4%, 126), White (73.6%, 120), and single (58.5%, 96).

**Table 1.** Demographics, and reproductive history of our study population.

Sex, % (n) Male, Female, % (n) Prefer not to say, % (n)	41.0 (68) 53.6 (89) 5.4 (9)
Gender Man, % (n) Woman, % (n) Non-binary, % (n) Prefer not to say, % (n) Other, % (n)	41.0 (68) 51.2 (85) 1.2 (2) 4.8 (8) 1.2 (2)
Age group 20–25 years, % (n) 26–30 years, % (n) 31–35 years, % (n) 36- 40 years, % (n) >40 years, % (n)	47.0 (77) 49.4 (81) 1.8 (3) 1.2 (2) 0.6 (1)
Sexual Orientation Heterosexual, % (n) Homosexual, % (n) Bisexual, % (n) Prefer not to say, % (n) Other, % (n)	76.4 (126) 5.5 (9) 8.5 (14) 4.8 (8) 4.8 (8)
Race White, % (n) Black, % (n) Hispanic, % (n) Asian, % (n) More than one race, % (n)	73.6 (120) 2.5 (4) 1.2 (2) 16.0 (26) 6.7 (11)
Religion Christian, % (n) Hindu, % (n) Jewish, % (n) Muslim, % (n) Atheist, % (n) Agnostic, % (n) Prefer not to say, % (n) Not specified, % (n)	43.2 (70) 4.3 (7) 3.0 (5) 1.8 (3) 6.8 (11) 9.3 (15) 10.5 (17) 21.1 (40)
Marital Status Married, % (n) Single, % (n) Divorced, % (n) Engaged, % (n)	23.8 (39) 58.5 (96) 1.8 (3) 15.9 (26)
Either participant or partner having been pregnant before, % (n)	11.3 (19)
Either participant or partner having had an abortion before, % (n)	0.6 (1)
Having children, % (n)	6.0 (10)
Plan to have children, Yes, % (n) No, % (n) Uncertain, % (n)	76.5 (127) 8.4 (14) 15.1 (25)

Regarding reproductive history, 19 students reported that either they or their partners had been pregnant before, while 10 students stated that they currently had children. Only 18 students voluntarily disclosed information about past abortions, with one student stating that they had undergone an abortion previously. Although a minority of students were parents, a significant proportion of them (76.5%, 127 students) expressed a desire to have or adopt children in the future.

### Medical students' views on Dobbs decision and abortion laws impact on medical education

Regarding the Supreme Court's *Dobbs* decision, a ruling that gave the states the authority to regulate abortion laws, 84.2% (75 students) of third-year and 79.7% (63) of fourth-year medical students disagreed with the decision. Additionally, 83.3% (140) of all student participants expressed disagreement with the current abortion laws in the state of Indiana. Although most of the students (71.4%, 120) did not consider state abortion laws when selecting a medical school, the consideration of these laws significantly increased when it came to applying for residency, with 66.3% (59) of third-year students and 40.3% (32) of fourth-year students indicating that they would take abortion laws into account when choosing a residency program. This signifies more than a threefold increase in the importance placed on abortion laws when applying for residency compared to medical school. 

When asked to prioritize factors for residency matching, the participants ranked location as the most important factor, followed by proximity to family and friends, with abortion being considered the first priority by four students. However, it was the among the top three priorities for 19.6% (33) of all students.

**Figure 1.**
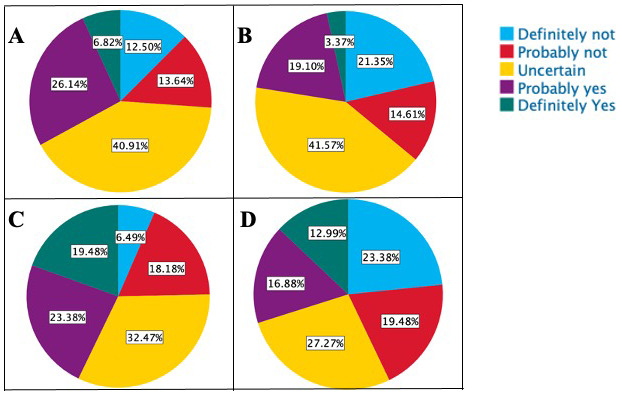
Impact of Dobbs Ruling on Medical Students’ Intention to Practice in Indiana After Completing Training. **A**. Plans of 3 ^rd^-Year Students **Before** to the Overturning of Roe v. Wade. **B**. Plans of 3 ^rd^-Year Students **After** the Overturning of Roe v. Wade. **C**. Plans of 4 ^th^-Year Students **Before** the Overturning of Roe v. Wade. **D**. Plans of 4 ^th^-Year Students **After** the Overturning of Roe v. Wade

*Third-year students (MS3s):* Out of the 89 MS3s who completed the survey, 24 expressed an interest in possibly pursuing an OB-GYN residency after medical school. While 12.4% (11) of MS3s stated that the *Dobbs* decision increased their interest in the specialty, another 25.8% (23) noted that it decreased their interest. Additionally, the majority (59.6%, 53 students) planned to apply and rank residency programs in states where abortion is legal, with only six students (6.7%) expressing a preference for states where abortion is illegal. Furthermore, we examined whether MS3s intended to stay in the state of Indiana to practice after their training, comparing their plans before the Supreme Court ruling with the present state. The analysis revealed a statistically significant decline in the number of MS3s planning to practice in Indiana, decreasing from 32.9% (29 students) to 22.5% (20 students) (p<0.001). ( Figure 1)

*Fourth-year students (MS4s):* 79 students completed the survey, out of which 7 (8.9%) mentioned that they would pursue OB-GYN after finishing medical school. Most students (82.3%, 65) stated that the current abortion laws did not affect their interest in this field. Irrespective of their chosen residency specialty, 40.5% (32) of MS4s stated that they would be ranking residency programs in states where abortion is legal, whereas only 2 students (2.6%) showed a preference for states where abortion is illegal. When examining the impact of the Supreme Court's ruling on abortion laws on MS4s' intention to practice in Indiana after their training, we found that while 42.9% (34) of MS4s had planned to practice in Indiana earlier, this sentiment declined to 29.9% (24) (p<0.001). ( Figure 1)

###  Demographic influences on personal beliefs and plans after the Dobbs decision

We also explored the influence of participant demographic data on their personal beliefs regarding abortion and the current abortion laws in Indiana, as well as their priorities when selecting residency training and practice locations.

*Sex and Gender:* There were identical patterns in both birth-assigned sex and self-identified gender among the participants. Hence, we described the impact of sex on our survey results (see summarized Table 2 for statistically significant observations). Females expressed significantly stronger disapproval of the current state abortion laws, including the *Dobbs* decision itself. They also considered abortion laws to be more important than males when deciding which states to apply for their residency programs. It is important to note that all the students in our cohort who were considering or applying to OB-GYN residency programs were females.

**Table 2.** Sex-based differences in Medical Students’ Views on the Dobbs Decision and its Influence on Career Preferences.

	Males	Females	P-value
Agreement with the overturning of Roe v Wade, % (n) Disagree Agree Neutral	73.5 (50) 25.0 (17) 1.5 (1)	95.5 (84) 3.4 (3) 1.1 (1)	<0.001 *
Satisfaction with Indiana laws about abortion, % (n) Dissatisfied Satisfied Neutral	75.0 (51) 23.5 (16) 1.5 (1)	94.3 (84) 5.6 (5) 0 (0)	<0.001 *
Abortion laws among the top three factors when considering residency programs, % (n)	7.5 (5)	33.7 (28)	<0.001 *
Considering OB-GYN residency, 3 ^rd^ years, % (n) Definitely yes May or may not Definitely not	0 (0) 5.1 (2) 94.9 (37)	27.1 (13) 16.6 (8) 56.3 (27)	<0.001 *
Consideration of abortion laws when applying for residency, 3 ^rd^ years , % (n) States with abortion LEGAL desirable States with abortion ILLEGAL desirable No impact	77.1 (37) 2.0 (1) 20.8 (10)	41.0 (16) 12.8 (5) 46.2 (18)	0.02 *
Applying to OB-GYN residency, 4 ^th^ years, % (n)	0 (0)	17.1 (7)	0.03 *
Consideration of abortion laws when ranking residency programs for residency, 4 ^th^ years, % (n) States with abortion LEGAL desirable States with abortion ILLEGAL desirable No impact	31.0 (9 0 (0) 20.8 (20)	53.7 (22) 0 (0) 46.3 (19)	<0.001 *

*denotes statistically significant *P*-value

*Marital Status:* Single students exhibited a higher level of dissatisfaction with the current abortion laws (89.5%, 86 students) compared to their married counterparts (66.7%, 26 students) or those who were engaged (80.7%, 21 students) (p = 0.043). Specifically, among fourth-year medical students who were single, a larger proportion expressed a preference for residency programs in states where abortion is legal compared to those who were engaged or married (67.9% vs. 21.6%, p = 0.029). However, these trends were not observed among third-year students in the study.

We also examined students' intentions regarding practicing medicine in Indiana before and after the *Dobbs* decision. While there was no statistically significant impact of marital status on fourth-year students' intention to practice in Indiana prior to the ruling, this factor became significant following the ruling. Notably since the change in abortion laws, single students were more inclined to opt out of practicing in Indiana after their training compared to their married peers (55.3% of single students vs. 29.6% of married students, p = 0.033).

*Previous Pregnancies and Having Children:* Students who themselves or their partners had experienced a previous pregnancy were more likely to agree with the *Dobbs* decision (36.8%, 7 students) compared to those who had never been pregnant (8.3%, 12 students, p = 0.006). Similarly, students who had experienced a previous pregnancy expressed greater satisfaction with the abortion laws in Indiana (31.5%, 6 students) compared to those who had never been pregnant (14.3%, 21 students, p = 0.02). Due to the limited number of participants who reported having children at present (10 participants), we were unable to draw meaningful comparisons regarding this factor in relation to the survey results.

*Age, Race, and Religion:* We found no significant impact of age and race on any of the survey questions. However, only students who identified as Christians (23 students, accounting for 32.9% of all Christian-identifying participants) expressed satisfaction with the current abortion laws in Indiana. In contrast, all participants who identified as Hindus, Muslims, Jews, Agnostic, or Atheist expressed dissatisfaction with the current abortion laws in Indiana (p = 0.06).

## Discussion

Our study revealed that four-fifths of Indiana University medical students in their clerkship years disagreed with the *Dobbs* decision. Importantly, while state abortion laws were not a consideration by our cohort when selecting medical schools, the post *Dobbs* era has led most students to factor in these laws when making decisions about their medical careers, including residency and practice choices.

A significant aspect of our study is the focus on rearticulating the cultural presentation of abortion, therefore we focused on women's experiences, agency, and decision-making processes. Historically, these perspectives have been influenced by viewpoints primarily from White males who cannot experience childbirth, thus leading to the persistence of biases. To address the culture of bias, it is crucial to shift the focus towards understanding the cultural presentation from the viewpoint of those who can bear children. Comparing the responses of males and females in our study, we found that females expressed significantly higher levels of dissatisfaction with the *Dobbs* decision. Moreover, they gave a much higher consideration to abortion laws when applying for any residency program, as evidenced by 38 out of 80 females in our study (47.5%) expressing a desire to train in programs where abortion is legal. Our data thus support the notion that female medical students are making career decisions impacted by abortion, considering not only the specialty they will be trained in (e.g., OB-GYN) but also how it may affect their maternal futures. By centering the experiences of women and acknowledging the various factors influencing their decisions, we can create a more nuanced and accurate cultural presentation of abortion ^18^. This approach will help us better understand the impact of societal structures on women's reproductive choices and how their decisions are influenced by personal aspirations and future considerations. It is also important to note that in our current cohort of fourth-year medical students, only women stated their interest in applying to OB-GYN residency, and only just two male MS3s were contemplating this career pathway. Given that women constitute the majority of obstetricians, it becomes even more significant to consider their experiences and insights when shaping policies and approaches related to abortion and reproductive healthcare.

The other component of structural competency we used for our analysis seeks to impart recognitions that the institutional structures that shape health are neither timeless nor immutable, but instead reflect legislative and cultural decisions made at a specific moment in time. When discussing legislative work such as *Dobbs v. Jackson*, women’s health now creates a narrative in which the dangers to those who can bear children in pre- *Roe vs. Wade* America may come to be the new norm. Religion is a well-known cultural influencer when it comes to personal and political decision-making. A 2020 metareview of 116 journal articles found that religion is by far the most utilized statistically significant independent variable when it comes to attitudes towards abortion ^19^. Another survey from 2022 of over 150,000 people in 76 countries found that for every increase in personal religious importance, people have a 0.491-unit increase in their disapproval of abortion ^20^. These data are consistent with our study since students who identified as Christian in our study were more likely to be satisfied with abortion restrictions. However, our study also highlights how all those who identify as other than Christian (namely Hindu, Muslim, Jewish, Agnostic, or Atheist) were dissatisfied with the current abortion restrictions in Indiana. Moreover, it must be acknowledged how the religious principles of some will have long lasting implications on women's health for years to come. Within our own United States 118 ^th^ Congress, 87.8% identify as Christian (compared to 63% of the general population) while only 6.2% identify as Jewish, 0.6% Muslim, 0.4% Hindu, and only 0.2% who identify as Unaffiliated (compared to 29% of the general population) ^21^.

As we consider the possible impact of the Dobbs decision on healthcare, our study reminds us of the importance of also assessing its impact on the healthcare workforce. Specifically, we show that specific demographics, particularly females and individuals with more liberal views, are more likely to leave impacted states like Indiana. This would mean that places most affected by this ruling could face a twofold challenge: firstly, patients facing obstacles in accessing reproductive services, and secondly, healthcare professionals in these areas may lean towards conservative views, potentially leading to a reluctance to provide such care ^22^. The medical education community must recognize this impact within affected states and take proactive steps to ensure that students receive relevant details on this complex subject. It is imperative to equip students with all the information necessary to comprehend the complexities surrounding abortion laws, empowering them to shape their beliefs and make informed decisions about their future careers and practice locations. This would allow students to approach their medical career decisions not from a standpoint of fear stemming from political climate, but with an understanding of the intricacies involved.

There are several strengths to our study. Firstly, this is one of the first studies designed to collect demographic data along with personal views on abortion and stratified the results accordingly. A study of this design allows for a more insightful exploration regarding the decision-making processes of our country’s future doctors about where they want to train and live. Another strength in our study is that the authors all work at Indiana University, the largest medical school in the country, and a medical institution within one of the first states to restrict abortion after the *Dobbs* decision. Not only did that give our survey participants the longest exposure on the intersection of politics and healthcare, but also provided the largest pool of third- and fourth-year medical students at one institution in the country.

There were also a few limitations to our study. The most obvious is a low response rate of 23.3%, which can be attributed to survey fatigue with the many survey asks our students receive. In addition, there is always potential in a voluntary survey for self-selection bias. Although the demographics represent our medical student body, opinions on abortion may have influenced whether students elected to participate in our survey. 

### Future Directions and Recommendations

When considering the results of this study, we have several recommendations for future research directions. Firstly, we recommend that future studies should focus on expanding the intersection of politics and healthcare, in particular continued expansion on studying abortion access and its effect on physician recruitment, both for training and for preferred location for living, as decreased rates of physician recruitment can worsen healthcare outcomes for the entire state. This is also true in states with abortion restrictions, as studies have shown that restriction to abortion worsens healthcare outcomes and studies should continue to document these outcomes. Along with this effort, medical institutions should create systems to keep track of work force demographics and the potential long-term impacts shifts in these demographics may have on both access to care and outcomes. We also recommend replication of studies like this across other medical institutions, particularly within restricted states, and qualitative studies to contextualize the findings.

Future efforts should continue at the institutional level to improve healthcare outcomes and maintain equal access to care, both for patients and for trainees. As stated previously, it is a requirement for U.S. OB-GYN residents and medical students to have access to abortion training. Institutions in states with abortion restrictions should formalize processes to provide access to such training for their learners and access to equitable healthcare for their state’s citizens.

## Conclusion

Our research highlights the impact of the Dobbs decision on how third- and fourth-year medical students at a large medical school in the United States make career choices. It emphasizes the role that abortion laws can have in shaping the landscape of healthcare workforce. By delving into the relationship between beliefs, structural factors and religious affiliations concerning abortion healthcare, we emphasize the need for a more nuanced understanding of how societal structures impact women's reproductive decisions and career paths in medicine. Further research is required to explore the intersection of healthcare and politics with regards to the impact of abortion access on trainee and physician recruitment and practice location decisions.

## Ethical approval and consent

The study was granted exempt status and approved by the Indiana University IRB, IRB Number: 17160. While a signed informed consent was not applicable to our study as determined by our IRB (Approval was obtained from our rigorous IRB before conducting any aspect of the study), we did provide all participants with a study information sheet (SIS). This served as an informed consent which did not need signature and stated if a participant agreed to participate, they will complete the survey. The extended data file which contains the survey (mentioned in manuscript and publicly available on OSF) has the SIS also attached which served as the consent form. As this study contains no identifiable information and was exempt per our Institutional Review Board, this was deemed sufficient for obtaining consent.

## Data Availability

OSF: A Matter of Choice: A Cross-section Study Examining the Impact of the Overturning of Roe v Wade on U.S. Medical Students’ Perceptions and Career Decisions. https://doi.org/10.17605/OSF.IO/TQ3WU ^23^ The project contains the following underlying data: Med students Perception of Roe V. Wade De_Ided data.xlsx OSF: A Matter of Choice: A Cross-section Study Examining the Impact of the Overturning of Roe v Wade on U.S. Medical Students’ Perceptions and Career Decisions. https://doi.org/10.17605/OSF.IO/TQ3WU ^23^ The project contains the following extended data: Appendix-Survey.pdf OSF: STROBE checklist for ‘A matter of choice: a cross-sectional study examining the impact of the overturning of Roe v Wade on U.S. medical students’ perceptions and career decisions’. https://doi.org/10.17605/OSF.IO/TQ3WU ^23^ Data are available under the terms of the Creative Commons Attribution 4.0 International license (CC-BY 4.0).

## References

[1] https://supreme.justia.com/cases/federal/us/410/113/. Accessed October 1st, 2023.

[2] https://supreme.justia.com/cases/federal/us/597/19-1392/. Accessed October 1st, 2023.

[3] https://www.supremecourt.gov/opinions/21pdf/19-1392_6j37.pdf. Accessed October 1st, 2023.

[4] ACOG committee opinion no. 613: increasing access to abortion. *Obstet Gynecol.* 2014;124(5):1060–1065. 10.1097/01.AOG.0000456326.88857.31 25437742

[5] APGO medical student educational objectives,11th edition. Accessed October 1st 2023. Reference Source

[6] Association of American Medical Colleges: Total enrollment by U.S. MD-granting medical school and gender, 2018-2019 through 2022-2023. Accessed October 1st 2023. Reference Source

[7] https://www.nrmp.org/match-data-analytics/residency-data-reports/. Accessed October 1st, 2023.

[8] https://www.guttmacher.org/2023/01/six-months-post-roe-24-us-states-have-banned-abortion-or-are-likely-do-so-roundup. Accessed October 1st, 2023.

[9] Fleszar LG Bryant AS Johnson CO : Trends in state-level maternal mortality by racial and ethnic group in the United States. *JAMA.* 2023;330(1):52–61. 10.1001/jama.2023.9043 37395772 PMC10318476

[10] Williams AM Chaturvedi R Pollalis I : Associations between state policies, race, ethnicity and rurality, and maternal mortality and morbidity following the United States supreme court dobbs v. jackson women's health organization ruling. *Br J Anaesth.* 2022;129(6):e145–e147. 10.1016/j.bja.2022.08.016 36163076

[11] https://www.acog.org/news/news-releases/2022/07/more-than-75-health-care-organizations-release-joint-statement-in-opposition-to-legislative-interference. Accessed October 1st, 2023.

[12] Stephenson-Famy A Sonn T Baecher-Lind L : The dobbs decision and undergraduate medical education: the unintended consequences and strategies to optimize reproductive health and a competent workforce for the future. *Acad Med.* 2023;98(4):431–435. 10.1097/ACM.0000000000005083 36347017

[13] Jones RK Jerman J : Population group abortion rates and lifetime incidence of abortion: United States, 2008-2014. *Am J Public Health.* 2017;107(12):1904–1909. 10.2105/AJPH.2017.304042 29048970 PMC5678377

[14] Levy MS Arora VM Talib H : Abortion among physicians. *Obstet Gynecol.* 2022;139(5):910–912. 10.1097/AOG.0000000000004724 35576350

[15] https://www.aclu-in.org/en/press-releases/indiana-supreme-court-rules-near-total-abortion-ban-can-take-effect. Accessed October 1st, 2023

[16] https://www.nbcnews.com/health/health-news/indiana-doctor-gave-10-year-old-girl-abortion-disciplinary-hearing-rcna86214. Accessed October 1st, 2023.

[17] Metzl JM Hansen H : Structural competency: theorizing a new medical engagement with stigma and inequality. *Soc Sci Med.* 2014;103:126–133. 10.1016/j.socscimed.2013.06.032 24507917 PMC4269606

[18] Osborne D Huang Y Overall NC : Abortion attitudes: an overview of demographic and ideological differences. *Polit Psychol.* 2022;43(S1):29–76. 10.1111/pops.12803

[19] Adamczyk A Kim C Dillon L : Examining public opinion about abortion: a mixed-methods systematic review of research over the last 15 years. *Sociol Inq.* 2020;90(4):920–954. 10.1111/soin.12351

[20] Adamczyk A : Religion as a micro and macro property: investigating the multilevel relationship between religion and abortion attitudes across the globe. *Eur Sociol Rev.* 2022;38(5):816–831. 10.1093/esr/jcac017

[21] https://www.pewresearch.org/religion/2023/01/03/faith-on-the-hill-2023/. Accessed October 1st, 2023.

[22] Wen DL Grossman LS Kopacz DR : Attitudes about abortion among second-year medical students. *Med Teach.* 1996;18(4):345–346. 10.3109/01421599609034191 11657032

[23] Saeed ZI Sharp S Conklin A : A matter of choice: a cross-section study examining the impact of the overturning of roe v wade on U.S. medical students’ perceptions and career decisions.2024. 10.17605/OSF.IO/TQ3WU PMC12375184 40860790

