# A matter of choice: a cross-sectional study examining the impact of the overturning of *Roe v Wade* on U.S. medical students’ perceptions and career decisions

**DOI:** 10.12688/mep.20519.2

**Published:** 2025-05-30

**Authors:** Alissa Conklin, Zeb Saeed, Sacha Sharp

**Affiliations:** 1 Department of Obstetrics and Gynecology, Indiana University School of Medicine, Indianapolis, Indiana, USA; 2 Department of Medicine, Indiana University School of Medicine, Indianapolis, Indiana, USA

**Keywords:** abortion, education, career choices

## Abstract

**Background:**

In June 2022, the *Dobbs* decision by the U.S. Supreme Court overturned federal abortion protections. In states with restrictive abortion laws such as Indiana, which also has the country’s largest medical school and the third worst maternal mortality rate, the impact of this ruling may have a significant impact on healthcare in the state. The purpose of this study was to analyze perceptions of medical students in Indiana in their third and fourth years of education after the *Dobbs* decision to assess if the state’s current abortion restrictions impact their career choice.

**Methods:**

Between December 2022 and March 2023, an anonymous survey was carried out at Indiana University School of Medicine, which included questions about personal beliefs on abortion and the current abortion laws in Indiana, as well as priorities when choosing residency training and practice locations.

**Results:**

Our survey found that four-fifths of medical students in Indiana disagreed with the *Dobbs* decision. While most students (71.4%) had not considered state abortion laws when selecting a medical school, since the Dobbs decision, 66.3% of third-year and 40.3% of fourth-year students indicated that they would take abortion laws into account when choosing a residency program. 47.5% of women students stated that they will be seeking residency in a state where abortion is legal and 55.3% of single students were more likely to leave Indiana to practice medicine.

**Conclusion:**

Our research suggests that physicians who are more liberal in their views on abortion may now be much less likely to practice in conservative states which will compound the healthcare outcomes secondary to the Dobbs decision. We emphasize the role that abortion laws have in shaping the landscape of healthcare workforce and the need for a more nuanced understanding of how societal structures impact women's reproductive decisions and career paths in medicine.

## Introduction

Abortion has historically been a contentious healthcare issue in the United States. In 1973, the U.S. Supreme Court ruled in Roe v. Wade that abortions are a right protected by the constitution, striking down several anti-abortion laws ^1^. Roe v Wade solidified a woman’s right to privacy and enshrined abortion healthcare as an essential aspect of women's health. However, in June 2022, the U.S. Supreme Court reversed that decision in *Dobbs v Jackson Women’s Health Organization,* stating the Constitution does not confer a right to abortion, thus returning the power to regulate aspects of abortion back to the individual states ^2, 3^. This ruling contradicts the shared decision-making efforts that had been established in women's healthcare. Moreover, the decision conflicted with the 2014 American College of Obstetricians and Gynecologists (ACOG) recommendations that abortion education be included in the curricula of all medical schools given states now held the power to decide whether abortion healthcare is legal ^4^. These conflicting priorities between the federal law and leading medical associations create challenges for medical schools in states where abortion restrictions limit reproductive healthcare education for medical school trainees.

The Association of Professors of Gynecology and Obstetrics (APGO) asserted, “Regardless of personal views about abortion, students should be knowledgeable about its public health importance, as well as techniques and patient safety implications” ^5^. It is also imperative to acknowledge that women are the demographic most significantly impacted by restrictive abortion laws. Presently, women are the largest group of matriculants into medical school with 53.8% of matriculating students in the 2022–2023 school year being women ^6^. Regarding matching into residency, 2022 match data show that of the 1,836 students who matched into OB/GYN residency training programs, 86.4% identify as women ^7^. In fact, OB/GYN as a specialty has the highest proportion of women applicants and matched students of all specialties in the country ^7^. Hence women medical students are doubly impacted by this ruling since potential laws not only restrict abortion healthcare, but could also limit a student’s access to comprehensive healthcare education. Beyond receiving training, all medical students and physicians, as well as their partners, are also patients. Access to their own adequate healthcare must be considered in the decision-making paradigm of life choices, including where to train and where to practice medicine. Almost one in four (23.7%) women in the United States will have an abortion by age 45, and medical training often takes place during a woman’s reproductive years ^8^. A 2021 survey of over 3,000 physicians and medical students who desired children found that 1 in 6 medical students or their partners had had an abortion ^9^.

It is estimated that 9,505 (41.9%) matriculating medical students will receive their medical education in the 24 states that have prohibited or severely restricted abortion since the *Dobbs* decision ^10^. One study utilizing the 2018–2020 state-level maternal mortality and morbidity from the Healthcare Cost and Utilization Project database found that per 100,000 births, the maternal mortality rate was more than double in states with restrictive laws on abortion compared to states with protective laws on abortion (4.0 vs 9.3) ^11^. Medical educators in these states are concerned that newly graduating medical students may not wish to get their residency training or to stay within a state that is restrictive towards abortion ^12, 13^. One state with restrictive abortion healthcare laws is Indiana. Indiana has the largest medical school in the country and the third highest maternal mortality rate in the country ^14^. The objective of this study was to analyze the perceptions of Indiana medical students in their third and fourth years of education after the *Dobbs* decision and to assess if the state’s current abortion restrictions impact their career choice, location for residency training, and where they ultimately desire to practice and live. Using these data, we hope to expand what we know about medical students' experiences with restrictive abortion healthcare and how that relates to career choice.

## Methods

Between December 2022 and March 2023, a cross-sectional survey was carried out among third- and fourth-year medical students at Indiana University School of Medicine (See Extended data section below). The survey was designed by all three authors together through an iterative process with multiple editions before finalizing the survey. At the time of its development in 2022, there were no validated instruments or similar surveys to emulate. Hence, we modeled several of our questions after the only publicly available survey at the time, a BestColleges' survey of undergraduate and graduate students ^15^. To identify relevant factors that may influence residency choices for students, we sought guidance from the 2021 NRMP Applicant Survey, especially, on data on applicants pursuing OB/GYN residency ^16^. To ensure the timeliness of this survey in the midst of changing laws, we decided to forego piloting the survey instead of delaying it.

The survey was anonymous and included questions about personal beliefs on abortion and the current abortion laws in Indiana, as well as priorities when choosing residency training and practice locations. The survey asked questions about the influence of abortion laws on the students’ decision-making and whether students intended to stay in Indiana, considering the recent *Dobbs* decision. Demographic information and variables including sex, gender, and religious affiliation was also collected to gather as social context. The survey was conducted in Qualtrics and distributed twice via a student newsletter and once via direct email to all third- and fourth-year medical students. The study was granted exempt status and approved by the Indiana University IRB, IRB Number: 17160. Survey data was exported into excel from Qualtrics and then to SPSS 28.0, which was used to analysis. Descriptive statistics (mean, percentage and frequencies) were used to describe the data. Chi-square was performed to examine the associations between various demographic factors and participant responses. A p-value of <0.05 was considered statistically significant.

We used structural competency to frame and interpret the data assessing medical students’ perceptions of the *Dobbs* decision and how it affects their career decisions. Within the competency framework, structures are defined as “policies, economic systems, and other institutions that have been produced and maintain social inequities and health disparities, often along the lines of social categories such as race, class, gender, and sexuality ^17^. Although our study used a cross-sectional survey which did not directly measure structural conditions, we interpreted students’ responses, particularly those identifying as women or those with the ability to give birth, those with identities marginalized by race, or sexual orientation within the broader context of how structural forces shape opportunity, agency, and choices in medical training. Restrictive abortion legislation is not only a health policy issue, but also a structural factor that may differentially affect women in medicine by limiting access to comprehensive reproductive care, limiting training opportunities in OB/GYN care, and influencing professional decisions around where to train or practice.

Additionally, we also reflected on our positionality as a team when analyzing and interpreting this data. All authors identify as cis-gender women. One of the research team members identifies as white, one identifies as Southeast Asian, and the final research member identifies as Black. The research team members share a passion and responsibility for educating and treating individuals from underrepresented backgrounds. We had multiple open discussions when interpreting the data to minimize any potential biases and ensure an objective interpretation.

## Results

A total of 763 third- and fourth-year IU medical students were invited to participate in the study. Of them, 178 students initiated the survey, resulting in a response rate of 23.3%. Out of those who started the survey, 168 students completed it and only these results were included in the final analysis. The demographic characteristics of the participants are summarized in Table 1 and are indicative of these classes’ heterogeneity. Though we could not collect demographic information of students who didn’t respond as would be outside of the scope of IRB, the distribution of sex and race in our cohort mirror publicly available data on IU school of medicine student body ^18^. Our cohort consisted of 53.6% (89) females, primarily individuals aged 30 or younger (96.4%,158), identifying as heterosexual (76.4%, 126), White (73.6%, 120), and single (58.5%, 96).

**Table 1.** Demographics, and reproductive history of Indiana University medical students who responded to survey on perceptions regarding abortion laws and career decisions.

Sex, % (n) Male, Female, % (n) Prefer not to say, % (n)	41.0 (68) 53.6 (89) 5.4 (9)
Gender Man, % (n) Woman, % (n) Non-binary, % (n) Prefer not to say, % (n) Other, % (n)	41.0 (68) 51.2 (85) 1.2 (2) 4.8 (8) 1.2 (2)
Age group 20–25 years, % (n) 26–30 years, % (n) 31–35 years, % (n) 36–40 years, % (n) >40 years, % (n)	47.0 (77) 49.4 (81) 1.8 (3) 1.2 (2) 0.6 (1)
Sexual Orientation Heterosexual, % (n) Homosexual, % (n) Bisexual, % (n) Prefer not to say, % (n) Other, % (n)	76.4 (126) 5.5 (9) 8.5 (14) 4.8 (8) 4.8 (8)
Race White, % (n) Black, % (n) Hispanic, % (n) Asian, % (n) More than one race, % (n)	73.6 (120) 2.5 (4) 1.2 (2) 16.0 (26) 6.7 (11)
Religion Christian, % (n) Hindu, % (n) Jewish, % (n) Muslim, % (n) Atheist, % (n) Agnostic, % (n) Prefer not to say, % (n) Not specified, % (n)	43.2 (70) 4.3 (7) 3.0 (5) 1.8 (3) 6.8 (11) 9.3 (15) 10.5 (17) 21.1 (40)
Marital Status Married, % (n) Single, % (n) Divorced, % (n) Engaged, % (n)	23.8 (39) 58.5 (96) 1.8 (3) 15.9 (26)
Either participant or partner having been pregnant before, % (n)	11.3 (19)
Either participant or partner having had an abortion before, % (n)	0.6 (1)
Having children, % (n)	6.0 (10)
Plan to have children, Yes, % (n) No, % (n) Uncertain, % (n)	76.5 (127) 8.4 (14) 15.1 (25)

Nineteen students reported that either they or their partners had been pregnant before, while 10 students were currently parents. Eighteen students disclosed information about past abortions, with one student stating that they had undergone an abortion previously. Although a minority of students were parents (10 students), a significant proportion of all students (76.5%, 127 students) expressed a desire to have or adopt children in the future.

### Medical students' views on Dobbs decision and abortion laws impact on medical education

84.2% (75 students) of third-year (MS3) and 79.7% (63) of fourth-year medical students (MS4s) expressed disagreement with the Dobb’s decision. Additionally, 83.3% (140) of all student participants disagreed with the current abortion laws in the state of Indiana. Though most of the students (71.4%, 120) did not consider state abortion laws when selecting a medical school, the consideration of these laws significantly increased when it came to applying for residency, with 66.3% (59) MS3s and 40.3% (32) MS4s indicating that they would take abortion laws into account when choosing a residency program. This signifies a threefold increase in the importance placed on abortion laws when applying for residency compared to medical school.

When asked to prioritize factors for residency matching, the participants ranked location as the most important factor, followed by proximity to family and friends, with abortion being considered the first priority by four students. However, it was the among the top three priorities of six possible factors for 19.6% (33) of all students.

**Figure 1.**
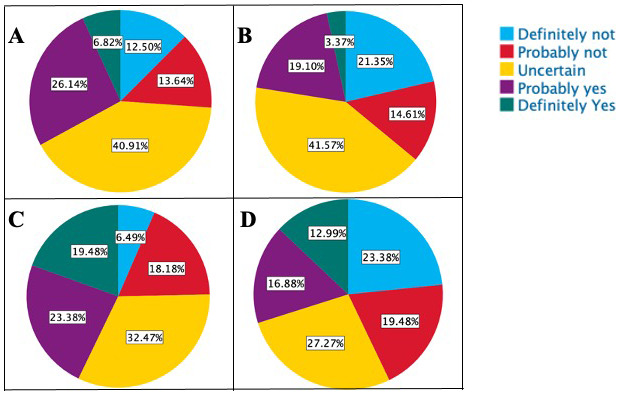
Impact of Dobbs Ruling on Medical Students’ Intention to Practice in Indiana After Completing Training. **A**. Plans of 3 ^rd^-Year Students **Before** the Overturning of Roe v. Wade. **B**. Plans of 3 ^rd^-Year Students **After** the Overturning of Roe v. Wade. **C**. Plans of 4 ^th^-Year Students **Before** the Overturning of Roe v. Wade. **D**. Plans of 4 ^th^-Year Students **After** the Overturning of Roe v. Wade.

*Third-year students (MS3s):* Out of the 89 MS3s who completed the survey, 24 expressed an interest in possibly pursuing an OB-GYN residency. 12.4% (11) of MS3s stated that the *Dobbs* decision increased their interest in the specialty, and 25.8% (23) noted that it decreased their interest. More students planned to apply and rank residency programs in states where abortion is legal (59.6%, 53 students) than those who preferred states where abortion was restricted (6.7%, 6 students). Additionally, MS3s identified that they were significantly less likely to practice in the State of Indiana after the overturning of Roe v Wade. ( Figure 1)

*Fourth-year students (MS4s):* 79 students completed the survey, out of which 7 (8.9%) mentioned that they would pursue OB-GYN after finishing medical school. Most students (82.3%, 65) stated that the current abortion laws did not affect their interest in this field. Independent of chosen residency, 40.5% (32) of MS4s reported that they would be ranking residency programs in states where abortion is legal, while 2 students (2.6%) expressed a preference for states where abortion is illegal. When examining the impact of Dobb’s decision on MS4s' intention to practice in Indiana after their training, we found that while 42.9% (34) of MS4s had planned to practice in Indiana earlier, this sentiment declined to 29.9% (24) (p<0.001) since the ruling. ( Figure 1)

### Demographic influences on personal beliefs and plans after the Dobbs decision

We also explored the influence of participant demographic data on their personal beliefs regarding abortion as well as their career priorities.

*Sex and Gender:* There were identical patterns in both birth-assigned sex and self-identified gender among the participants. We chose to describe the impact of sex on our survey results they were identical to those of gender and to examine the impact on those with the ability to give birth. (see summarized Table 2 for statistically significant observations). Women expressed significantly stronger disapproval of the current state abortion laws and were more likely to consider abortion laws when applying for residency programs. All the students in our cohort who were considering or applying to OB-GYN residency programs were women.

**Table 2.** Sex-based differences in Medical Students’ Views on the Dobbs Decision and its Influence on Career Preferences.

	Males	Females	P-value
Agreement with the overturning of Roe v Wade, % (n) Disagree Agree Neutral	73.5 (50) 25.0 (17) 1.5 (1)	95.5 (84) 3.4 (3) 1.1 (1)	<0.001 *
Satisfaction with Indiana laws about abortion, % (n) Dissatisfied Satisfied Neutral	75.0 (51) 23.5 (16) 1.5 (1)	94.3 (84) 5.6 (5) 0 (0)	<0.001 *
Abortion laws among the top three factors when considering residency programs, % (n)	7.5 (5)	33.7 (28)	<0.001 *
Considering OB-GYN residency, 3 ^rd^ years, % (n) Definitely yes May or may not Definitely not	0 (0) 5.1 (2) 94.9 (37)	27.1 (13) 16.6 (8) 56.3 (27)	<0.001 *
Consideration of abortion laws when applying for residency, 3 ^rd^ years , % (n) States with abortion LEGAL desirable States with abortion ILLEGAL desirable No impact	77.1 (37) 2.0 (1) 20.8 (10)	41.0 (16) 12.8 (5) 46.2 (18)	0.02 *
Applying to OB-GYN residency, 4 ^th^ years, % (n)	0 (0)	17.1 (7)	0.03 *
Consideration of abortion laws when ranking residency programs for residency, 4 ^th^ years, % (n) States with abortion LEGAL desirable States with abortion ILLEGAL desirable No impact	31.0 (9) 0 (0) 20.8 (20)	53.7 (22) 0 (0) 46.3 (19)	<0.001 *

*denotes statistically significant *P*-value

*Marital Status:* Single students exhibited a higher level of dissatisfaction with the current abortion laws (89.5%, 86 students) compared to students who were married (66.7%, 26 students) or engaged (80.7%, 21 students) (p = 0.043). Among MS4s, a larger proportion of single students expressed a preference for residency programs in states where abortion is legal compared to those who were engaged or married (67.9% vs. 21.6%, p = 0.029). However, these trends were not observed among MS3s in the study.

Similarly, single MS4s were more inclined to opt out of practicing in Indiana after their training compared to their married peers (55.3% of single students vs. 29.6% of married students, p = 0.033).

*Previous Pregnancies and Having Children:* Students who themselves or their partners had experienced a previous pregnancy were more likely to agree with the *Dobbs* decision (36.8%, 7 students) compared to those who had never been pregnant (8.3%, 12 students, p = 0.006). Similarly, students who had experienced a previous pregnancy expressed greater satisfaction with the abortion laws in Indiana (31.5%, 6 students) compared to those who had never been pregnant (14.3%, 21 students, p = 0.02). Due to the limited number of participants who reported having children at present (10 participants), we were unable to draw meaningful comparisons regarding this factor in relation to the survey results.

*Age, Race, and Religion:* We found no significant impact of age and race on any of the survey questions. However, all students who reported satisfaction with current abortion laws in Indiana had identified as Christians (23 students, accounting for 32.9% of all Christian-identifying participants). In contrast, all participants who identified as Hindus, Muslims, Jews, Agnostic, or Atheist expressed dissatisfaction with the current abortion laws in Indiana (p = 0.06 for satisfaction rates).

## Discussion

Our study revealed that 84.2% of third-year and 79.7% of fourth-year medical students at Indiana University disagreed with the *Dobbs* decision. Importantly, while state abortion laws were not a consideration by our cohort when selecting medical schools, the post *Dobbs* era has led most students to factor in these laws when making decisions about their medical careers, including residency and practice choices.

Our study focused on analyzing perspectives on abortion healthcare in relation to sex, gender, age, whether the participant or their partner has been pregnant, desire to have children and other demographics and priorities. Historically, perspectives on abortion have been influenced by viewpoints primarily from males who are white who cannot experience childbirth, thus leading to the persistence of biases. To address the culture of bias, it is crucial to shift the focus towards understanding the cultural presentation from the viewpoint of those who can bear children.

Comparing the responses of those who have the potential to become pregnant to those who do not in our study, we found that those who have the potential to become pregnant expressed significantly higher levels of dissatisfaction with the *Dobbs* decision. Moreover, they gave a much higher consideration to abortion laws when applying for residency programs. Our data thus support the notion that medical students with the potential to become pregnant are making career decisions impacted by abortion laws, considering not only the specialty they will be trained in (e.g., OB-GYN) but also how it may affect their reproductive futures. This may be based on protecting their reproductive autonomy and access to miscarriage and abortion care.

By centering the experiences of women in a culture and system that traditionally does not, e.g. a patriarchal society, and acknowledging the various factors influencing their decisions, we can create a more nuanced and accurate cultural presentation of abortion ^19^. It is also important to note that in our current cohort of fourth-year medical students, only women stated their interest in applying to OB-GYN residency, and only just two men MS3s were contemplating this career pathway. Given that women constitute the majority of obstetricians, it becomes even more significant to consider their experiences and insights when shaping policies and approaches related to abortion and reproductive healthcare.

Religion, a well-known cultural influencer when it comes to personal and political decision-making, is also important to consider in analyses regarding healthcare decisions and training. A 2020 metareview of 116 journal articles found that religion is by far the most utilized statistically significant independent variable when it comes to attitudes towards abortion ^20^. These data are consistent with our study since students who identified as Christian in our study were more likely to be satisfied with abortion restrictions. However, our study also highlights how all those who identify as other than Christian (namely Hindu, Muslim, Jewish, Agnostic, or Atheist) were dissatisfied with the current abortion restrictions in Indiana and their perspectives may be inadequately represented in positions of power among our country’s lawmakers. For example, within our own United States 118 ^th^ Congress, the Congress active at the time of this paper and responsible for representing the United States citizens and their priorities, the division of religious affiliation does not match the general population: 87.8% identify as Christian (compared to 63% of the general population) while only 6.2% identify as Jewish, 0.6% Muslim, 0.4% Hindu, and only 0.2% who identify as Unaffiliated (compared to 29% of the general population) ^21^. This leads to bias within our laws towards Christian values and beliefs even though the citizens of the United States have more varied religious perspectives.

As we consider the possible impact of the Dobbs decision on the healthcare workforce and medical education, it is important to understand the implications of these laws for both personal and professional access and training. In our study, we show that specific demographics, particularly females and individuals with more liberal views, are more likely to leave states with restrictive abortion laws like Indiana. This would mean that places most affected by this ruling could face a twofold challenge: firstly, patients facing obstacles in accessing reproductive services, and secondly, healthcare professionals in these areas may be more likely to be reluctant at providing such care and less likely to receive such training. Currently, 36% of all US counties lack an OB-GYN, and by 2030 there will be a projected overall deficit of 5,170 full-time equivalent (FTEs) OB-GYNs in this country ^22, 23^. The medical education community must recognize this impact and take proactive steps to ensure that students receive relevant details and access to training on the complex subject of abortion healthcare and the law. This type of proactive planning would empower students to approach their medical career decisions not from a standpoint of fear around a political climate, but with an understanding of the intricacies involved and options for both training and reproductive healthcare access for themselves and their patients.

There are several strengths to our study. Firstly, this study assessed personal views on abortion and stratified those results along demographic data. A study of this design allows for a more insightful exploration regarding the decision-making processes of our country’s future doctors about where they want to train and live. Another strength in our study is that the students surveyed attend the largest medical school in the country, and a medical institution within one of the first states to restrict abortion after the *Dobbs* decision. Not only did that give our survey participants the longest exposure on the intersection of politics and healthcare but also provided the largest pool of third- and fourth-year medical students accessible at a single institution.

There were also several limitations to our study. The most obvious is a low response rate of 23.3%, which can be attributed to survey fatigue given our students are often asked to complete many surveys throughout their education. In addition, there is always potential in a voluntary survey for self-selection bias. Although the demographics in our study accurately represent our medical student body, opinions on abortion healthcare may have influenced whether students elected to participate in our survey. While the students surveyed are at the largest medical school in the country, we only surveyed one medical school in a state with restrictive laws on abortion. Not all people who can bear children or need abortion care identify as women. And while there are some studies that assess nonbinary, transgender and gender-diverse populations and abortion care, 7.2% of our study participants identified as neither man nor woman, and future studies should assess this demographic more thoroughly. Lastly, given religious affiliation was associated with difference in perceptions regarding abortion laws, it would have been more meaningful to have additionally asked participants regarding their religious importance ^24^.

### Future directions and recommendations

When considering the results of this study, we have several recommendations for future research directions. Firstly, we recommend that future studies should focus on expanding the intersection of politics and healthcare, in particular continued expansion on studying abortion access and its effect on physician recruitment, both for training and for preferred location for living, as decreased rates of physician recruitment can worsen healthcare outcomes for the entire state. This is also true in states with abortion restrictions, as studies have shown that restriction to abortion worsens healthcare outcomes and studies should continue to document these outcomes. Along with this effort, medical institutions should create systems to keep track of workforce demographics and the potential long-term impacts that shifts in these demographics may have on both access to care and outcomes. We also recommend replication of studies like this across other medical institutions, particularly within restricted states, and qualitative studies to contextualize the findings.

Future efforts should continue at the institutional level to improve healthcare outcomes and maintain equal access to care, both for patients and for trainees. For patients, access to abortion and comprehensive reproductive services should be considered a healthcare disparity, considering half (49%) of those who have an abortion have incomes below the federal poverty line and the majority (62%) are nonwhite ^8, 25, 26^. Future studies should analyze data through the lens of access to reproductive healthcare and these disparities.

## Conclusion

Our research highlights the impact of the Dobbs decision on how third- and fourth-year medical students at a large medical school in the United States make career choices. It emphasizes the role that abortion laws can have in shaping the landscape of healthcare workforce. By delving into the relationship between beliefs, structural factors and religious affiliations concerning abortion healthcare, we emphasize the need for a more nuanced understanding of how societal structures impact women's reproductive decisions and career paths in medicine. Further research is required to explore the intersection of healthcare and politics with regards to the impact of abortion access on trainee and physician recruitment and practice location decisions.

## Ethical approval and consent

The study was granted exempt status and approved by the Indiana University IRB, IRB Number: 17160. While a signed informed consent was not applicable to our study as determined by our IRB (Approval was obtained from our rigorous IRB before conducting any aspect of the study), we did provide all participants with a study information sheet (SIS). This served as an informed consent which did not need signature and stated if a participant agreed to participate, they will complete the survey. The extended data file which contains the survey (mentioned in manuscript and publicly available on OSF) has the SIS also attached which served as the consent form. As this study contains no identifiable information and was exempt per our Institutional Review Board, this was deemed sufficient for obtaining consent.

## Data Availability

OSF: A Matter of Choice: A Cross-section Study Examining the Impact of the Overturning of Roe v Wade on U.S. Medical Students’ Perceptions and Career Decisions. https://doi.org/10.17605/OSF.IO/TQ3WU ^23^ The project contains the following underlying data: Med students Perception of Roe V. Wade De_Ided data.xlsx OSF: A Matter of Choice: A Cross-section Study Examining the Impact of the Overturning of Roe v Wade on U.S. Medical Students’ Perceptions and Career Decisions. https://doi.org/10.17605/OSF.IO/TQ3WU ^23^ The project contains the following extended data: Appendix-Survey.pdf OSF: STROBE checklist for ‘A matter of choice: a cross-sectional study examining the impact of the overturning of Roe v Wade on U.S. medical students’ perceptions and career decisions’. https://doi.org/10.17605/OSF.IO/TQ3WU ^23^ Data are available under the terms of the Creative Commons Attribution 4.0 International license (CC-BY 4.0).

## References

[1] Roe v. Wade | 410 U.S. 113 (1973) | Justia U.S. Supreme Court Center.Vol. 2023. Reference Source 12038376

[2] Dobbs v. Jackson Women's Health Organization | 597 U.S. ___ (2022) | Justia U.S. Supreme Court Cente.Vol. 2023. Reference Source

[3] DOBBS, STATE HEALTH OFFICER OF THE MISSISSIPPI DEPARTMENT OF HEALTH, ET AL. v. JACKSON WOMEN’S HEALTH ORGANIZATION ET AL. 2021. Reference Source

[4] ACOG Committee Opinion No. 613: increasing access to abortion. *Obstet Gynecol.* 2014;124(5):1060–1065. 10.1097/01.AOG.0000456326.88857.31 25437742

[5] APGO medical student educational objectives, 11th edition.Vol. 2023. Reference Source

[6] Association of American Medical Colleges: Total enrollment by U.S. MD-granting medical school and gender, 2018-2019 through 2022-2023.Vol. 2023.

[7] Match Data | NRMP.Vol. 2023. Reference Source

[8] Jones RK Jerman J : Population group abortion rates and lifetime incidence of abortion: United States, 2008–2014. *Am J Public Health.* 2017;107(12):1904–1909. 10.2105/AJPH.2017.304042 29048970 PMC5678377

[9] Levy MS Arora VM Talib H : Abortion among physicians. *Obstet Gynecol.* 2022;139(5):910–912. 10.1097/AOG.0000000000004724 35576350

[10] Six Months Post-Roe, 24 US States Have Banned Abortion or are likely to Do So/ A Roundup | Guttmache.Vol. 2023. Reference Source

[11] Williams AM Chaturvedi R Pollalis I : Associations between state policies, race, ethnicity and rurality, and maternal mortality and morbidity following the United States Supreme Court *Dobbs v. Jackson Women's Health Organization* ruling. *Br J Anaesth.* 2022;129(6):e145–e147. 10.1016/j.bja.2022.08.016 36163076

[12] More than 75 health care organizations release joint statement in opposition to Legislative Interfer.Vol. 2023. Reference Source

[13] Stephenson-Famy A Sonn T Baecher-Lind L : The dobbs decision and undergraduate medical education: the unintended consequences and strategies to optimize reproductive health and a competent workforce for the future. *Acad Med.* 2023;98(4):431–435. 10.1097/ACM.0000000000005083 36347017

[14] Fleszar LG Bryant AS Johnson CO : Trends in state-level maternal mortality by racial and ethnic group in the United States. *JAMA.* 2023;330(1):52–61. 10.1001/jama.2023.9043 37395772 PMC10318476

[15] Roe v. Wade ruling impacts College Choice for 39% of prospective students | BestColleges.Vol. 2025. Reference Source

[16] Results of the 2021 NRMP applicant survey | NRMP.Vol. 2025. Reference Source

[17] Metzl JM Hansen H : Structural competency: theorizing a new medical engagement with stigma and inequality. *Soc Sci Med.* 2014;103:126–133. 10.1016/j.socscimed.2013.06.032 24507917 PMC4269606

[18] Class Profile - Demographics of IU Indianapolis Students.Vol. 2025. Reference Source

[19] Osborne D Huang Y Overall NC : Abortion attitudes: an overview of demographic and ideological differences. *Polit Psychol.* 2022;43(S1):29–76. 10.1111/pops.12803

[20] Adamczyk A Kim C Dillon L : Examining public opinion about abortion: a mixed-methods systematic review of research over the last 15 years. *Sociological Inquiry.* 2020;90(4):920–954. 10.1111/soin.12351

[21] The religious composition of the 118th congress | Pew Research Center.Vol. 2023. Reference Source

[22] Projections of supply and demand for women’s health service providers: 2018-2030. U.S. Department of health and human services, health resources and services administration, national center for health workforce analysis. 2021;2025. Reference Source

[23] Nowhere to Go/ Maternity care deserts across the US | March of Dimes.Vol. 2025. Reference Source

[24] Moseson H Fix L Ragosta S : Abortion experiences and preferences of transgender, nonbinary, and gender-expansive people in the United States. *Am J Obstet Gynecol.* 2021;224(4):376.e371–376.e311. 10.1016/j.ajog.2020.09.035 32986990 PMC7518170

[25] Jerman J Jones RK Onda T : Characteristics of U.S. abortion patients in 2014 and changes since 2008. 2016;2025. Reference Source

[26] Watson K : The ethics of access: reframing the need for abortion care as a health disparity. *Am J Bioeth.* 2022;22(8):22–30. 10.1080/15265161.2022.2075976 35621314

