# Supplementary material for: A matter of choice: a cross-sectional study examining the impact of the overturning of Roe v Wade on U.S. medical students’ perceptions and career decisions
Source: MedEdPublish (2016). 2025 May 30;14:64. Originally published 2024 Aug 16. [Version 2] doi: 10.12688/mep.20519.2 (PMC12375184; doi:10.12688/mep.20519.2)
Supplement: Peer review history

# Reviewer report: Reviewer response for version 2

**DOI:** 10.21956/mep.22549.r42002

**Published:** 2025-08-23

**Authors:** Jody Steinauer

**Affiliations:** 1 Department of Obstetrics, Gynecology and Reproductive Sciences, University of California San Francisco, San Francisco, California, USA

It is much improved!  My main concern is that it is still quite wordy but I will defer to the publisher on their perspectives about whether it should be shortened.

 I just have a few clarifying questions.

 In results: "Eighteen students disclosed information about past abortions, with one student stating they had undergone an abortion previously." I'm confused about that - 19 - they or partner previously pregnant, 10 parents, 18 abortions, 1 abortion? This is confusing.

 In abstract: In the results section of your abstract you give us proportions but don't tell us what they mean.  Is the proportion of women who want to go to a state with legal abortion more than men?  Maybe say something instead like - Women were more likely than men to seek residency in a state where abortion is legal (xx% v. xx%, p=). ]By the way - where did the 47.5% come from? That's in the abstract but i don't see it anywhere else.  Do you mean 54% (v. 31% men?) That's in the table.]  Please double check all your numbers in the text and tables.

 In conclusion in the abstract: "Our research suggests that physicians who are more liberal in their views on abortion may now be much less likely to practice in conservative states which will compound the healthcare outcomes secondary to the Dobbs decision."  This seems to be a big punchline but it is not in the results section of your abstract.  (it is also hard to find in results and discussion. If this is so important maybe emphasize it more.) In the abstract you give us data about women but not about abortion beliefs. Maybe remove the single status and focus on sex and abortion attitudes as your 3rd sentence?  then in conclusion you can also mention women.

Have any limitations of the research been acknowledged?

Yes

Is the work clearly and accurately presented and does it cite the current literature?

Partly

If applicable, is the statistical analysis and its interpretation appropriate?

Partly

Are all the source data underlying the results available to ensure full reproducibility?

Partly

Is the study design appropriate and is the work technically sound?

Partly

Are the conclusions drawn adequately supported by the results?

No

Are sufficient details of methods and analysis provided to allow replication by others?

Partly

Reviewer Expertise:

Medical education, abortion care, abortion training, obstetrics and gynecology training, qualitative and quantitative research methods

I confirm that I have read this submission and believe that I have an appropriate level of expertise to confirm that it is of an acceptable scientific standard, however I have significant reservations, as outlined above.

# Reviewer report: Reviewer response for version 2

**DOI:** 10.21956/mep.22549.r42290

**Published:** 2025-08-23

**Authors:** Martha F. Davis

**Affiliations:** 1 Northeastern University, Boston, Massachusetts, USA

This article describes the results of a survey administered to med school students at Indiana University to determine the impact of the U.S. Supreme Court's decision in Dobbs on their future plans for med school education and medical practice.

 I commend the authors for undertaking this study and for publishing the results.  I do have some comments that I believe would improve the paper.

 In the "Results" section, the description of the results should clarify that the survey did not reach all medical students, or even all med students at Indiana.  I suggest that the summary refer to "respondents" to take account of the incompleteness of the data.

 In the Introduction, paragraph 1 refers to "shared decisionmaking."  In the context of the article, it's not clear what this refers to.  The article's description of Roe did not emphasize this aspect of the decision, and the authors might be referring to federal-state sharing rather than doctor-patient sharing.

 In paragraph 2, a brief description of US medical education would be very helpful.  In particular, many readers may not be familiar with the "match."  Indeed, the nature of the match may affect the interpretation of the results here, since med students don't simply select a program but have to prioritize several.  For each program they list, the balance of factors may be different.

 In the section titled "Medical students . . . views on the Dobbs decision":  it is very pertinent here that the students surveyed were selecting their med schools when there was a federal right, and state laws were less significant.  That explains the great difference between considerations for med school selection (pre-2022) and residency selection (post-2022).  This is implicit, but you really should spell it out.  Otherwise, it seems that maybe you're making a generalization about how students select med schools vs. residencies, when in fact, the ground shifted during the relevant time frame.

 In the 2d line of the 2d paragraph under "Demographic Influences," there are some words missing.  The piece reads "survey results they were . . . "

 Under the Discussion section:  again, I believe that you should refer to respondents here rather than make a claim about all med school students.  And as described above, the 2d paragraph doesn't make note of the different legal regimes when students were selecting med schools (past) vs. residencies (future).

 The 2d paragraph in the Discussion section asserts that abortion law/policy has been largely developed by white men.  This needs a cite of some kind.  You could cite to the Dobbs dissent for this, where the dissenting justices note that much of the history cited by the majority occurred before women could vote or hold property.

 In para. 5 of this section, you describe the demographics/religious affiliations of U.S. Congress.  But I'm not sure that's relevant to your points here.  Dobbs was a decision of the Supreme Court -- you might note the dominance of Catholics in the majority opinion, or you could tie the Supreme Court's membership to the judicial nomination process, but just citing religions in Congress doesn't connect the dots.  After Dobbs, abortion policy in Indiana is in the hands of the state courts and the state legislature, so Congress is only relevant if there's a push to establish a federal abortion law of one kind of another.  You don't address that possibility at all, and it was not part of the survey that you conducted.

Have any limitations of the research been acknowledged?

Yes

Is the work clearly and accurately presented and does it cite the current literature?

Partly

If applicable, is the statistical analysis and its interpretation appropriate?

I cannot comment. A qualified statistician is required.

Are all the source data underlying the results available to ensure full reproducibility?

Yes

Is the study design appropriate and is the work technically sound?

Yes

Are the conclusions drawn adequately supported by the results?

Partly

Are sufficient details of methods and analysis provided to allow replication by others?

Yes

Reviewer Expertise:

law

I confirm that I have read this submission and believe that I have an appropriate level of expertise to confirm that it is of an acceptable scientific standard, however I have significant reservations, as outlined above.

# Reviewer report: Reviewer response for version 2

**DOI:** 10.21956/mep.22549.r42284

**Published:** 2025-07-24

**Authors:** Mary Higgins

**Affiliations:** 1 University College Dublin (UCD) Perinatal Research Centre, Dublin, Ireland

Thank you for this very interesting and topical paper. My comments are intended to help further improve your work. 

**Abstract**

**-** Background - could you clarify if the medical students are in third/fourth year of medical school or third/fourth year since the Dobbs decision (given the timing of the decision presumably the former, but this is not clear). 

 -Demographic information would be useful in the abstract for context as would be some information about how the survey was developed. 

 Introduction 

 As someone who does not practice in the USA, why would education be affected  - do you mean that students would not get exposure or that the curriculum of knowledge would be affected?

  - I am interested in what percentage of students would normally stay in Indiana to practice before Dobbs, if you have any reference to this

 (Small point, but would suggest not starting sentences with numbers or percentages, and writing numbers less than ten as words ie one in six not 1 in 6) 

 -Writing the introduction as problem (restriction, population, education) gap and hook may be an option to further clarify and communicate. 

 Methods 

 I would like significantly more information on how the survey was developed and tested. Reference to the AMEE guide would be very useful. https://www.tandfonline.com/doi/full/10.3109/0142159X.2014.889814. I appreciate the context of background of the researchers and wonder if you could have considered including a student in the team with a PPI role. 

 Why pick third and fourth year students? why not just fourth years if they are about to graduate? (this may be context specific but specifying timing of residency applications would help with international understanding). What is the denominator (how many students were invited to

 How did you test for normality? (use of "mean" only suggests normally distributed data). 

 Results

 Please add "years" to "aged 30 or younger. 

 Suggest (96.4% n=158)

 I am interested in whether the students would consider changing states for residency because of practice implications or allowance for options in their personal lives. 

 MS3 - presumably means third year medical student?

 For previous pregnancies, the small numbers may affect a chi squared analysis (but noted all >5)

 The satisfaction rate is not statistically significant?

 Discussion 

 can you give a reference to support the sentence that historically perspectives on abortion are primarily from males? 

 Suggest rewriting the phrase "By centering the experiences of women in a culture and system that traditionally does not, e.g. a patriarchal society, and acknowledging the various factors influencing their decisions.."

 Within the demographic information requested, could it be possible to identify students? 

Have any limitations of the research been acknowledged?

No

Is the work clearly and accurately presented and does it cite the current literature?

Yes

If applicable, is the statistical analysis and its interpretation appropriate?

Partly

Are all the source data underlying the results available to ensure full reproducibility?

Partly

Is the study design appropriate and is the work technically sound?

Partly

Are the conclusions drawn adequately supported by the results?

Yes

Are sufficient details of methods and analysis provided to allow replication by others?

Yes

Reviewer Expertise:

Academic obstetrician

I confirm that I have read this submission and believe that I have an appropriate level of expertise to confirm that it is of an acceptable scientific standard, however I have significant reservations, as outlined above.

# Reviewer report: Reviewer response for version 2

**DOI:** 10.21956/mep.22549.r42000

**Published:** 2025-06-17

**Authors:** Helena Prior Filipe

**Affiliations:** 1 Egas Moniz Interdisciplinary Research Centre (Ringgold ID: 510440), Caparica, Setúbal, Portugal; 2 Ophthalmology, Hospital Centre of West Lisbon Campus Hospital of Egas Moniz, Lisboa, Lisbon, Portugal

Congratulations to the authors for their significantly constructive review of the manuscript. The writing flow has improved, inviting a full and thorough read. Assertions are systematically supported by evidence and argument. This manuscript's progression is instructive for both authors and reviewers and has now earned a place for indexing. I believe it will attract attention from MedEdPublish readership and generate valuable conversation.

Have any limitations of the research been acknowledged?

Partly

Is the work clearly and accurately presented and does it cite the current literature?

Yes

If applicable, is the statistical analysis and its interpretation appropriate?

Partly

Are all the source data underlying the results available to ensure full reproducibility?

Yes

Is the study design appropriate and is the work technically sound?

Partly

Are the conclusions drawn adequately supported by the results?

Yes

Are sufficient details of methods and analysis provided to allow replication by others?

Partly

Reviewer Expertise:

Mentorship, Faculty development with an emphasis for Social learning and Arts and Humanities, Online competency-based education and learning by Simulation, Continuing Professional Education

I confirm that I have read this submission and believe that I have an appropriate level of expertise to confirm that it is of an acceptable scientific standard.

# Reviewer report: Reviewer response for version 1

**DOI:** 10.21956/mep.21961.r41011

**Published:** 2025-02-26

**Authors:** Erica Cahill, Amythis Soltanti

**Affiliations:** 1 Stanford University, Stanford, USA; 2 Gynecology Department, Stanford University Department of Obstetrics and Gynecology (Ringgold ID: 196271), Stanford, California, USA

The purpose of this study was to analyze perceptions of medical students at Indiana University School of Medicine in their third and fourth years of education after the Dobbs decision to assess if the state’s current abortion restrictions impact their career choice through an anonymous survey sent between December 2022 and March 2023. The results showed that people with liberal views on abortion care are more likely to want to practice in states with less restrictive abortion laws, which can continue to create further disparities in access to abortion care for people living in these states. 

 This is an important topic and the authors clearly justify why medical students in Indiana are a good specific test scenario (largest med school, restrictive laws, maternal mortality rates). However, we have some concerns about the low response rate (23%) which makes it difficult to draw conclusions about impact on medical student careers. More details on non-responders/the entire 3rd/4th year demographics would be helpful in clarifying if this is a representative sample. The authors do say this in their discussion but do not give specific data to clearly support this. Additionally there is significant information missing in the methods section including outcomes of interest, how missing data was approached, and what answer choices were given as options (e.g. in priorities for residency match, the authors do not specify how many options were given before saying 19% chose abortion laws in their top 3).Recommend referring to STROBE checklist to make sure all methodology is included in report. (https://www.strobe-statement.org/checklists/). The conclusions must be adapted to the limitations of the study. In this current version, these conclusions are too strong/certain for what is presented. 

**Abstract**

- For background, clarify what "the impact of this ruling may be huge on the medical field" -link to this topic specifically, as in "it is unknown what the impact of this ruling is on medical student career choices in Indiana"
- Results: Add 23% response rate to Results section as first sentence. then say, of the students who responded...
- Use percents instead of fractions (e.g. four-fifths)
- Conclusion: Be careful about using terms like liberal in regards to abortion views. Be more specific on what you can state based on the questions you asked

**Introduction**

- Consider removing “went one step further” in the following sentence: In addition, the Association of Professors of Gynecology and Obstetrics (APGO) went one step further and asserted, “Regardless of personal views about abortion, students should be knowledgeable about its public health importance, as well as techniques and patient safety implications” 5. 
  - Consider changing abortion to abortion care where applicable throughout the manuscript.
  - Unclear how reference 15 is being used where it is cited. Please provide further clarification.
  - Consider rephrasing “In the case of Dr. Caitlin Bernard, the nation has witnessed how a physician can be scrutinized and reprimanded for doing their job, and how abortion law restrictions and political motivations can disrupt work and life 16. “ → from “doing their job” to “providing abortion care”. Also not every reader will be familiar with this case, therefore it may be reasonable to provide further clarification. 
  - Decide on wording women or females and be consistent. Prefer women as female is used as a descriptor for animals (e.g. female mouse)
- **Methods**
  - an outcome is not specified
  - Please refer to the STROBE checklist to make sure you're including all important information including: variables, methods of assessment, and statistical methods for data analysis. How was missing data analyzed for the students who started but did not complete entire survey? Were there responses included?
- **Results **
  - It would be helpful to compare respondents demographics to non-respondent demographics (or to the whole 3rd and 4th year demographics) to show that it is in fact representative (adapt Table 1 to include a second column representing the entire 3rd/4th year class with as much information as you have. 
  - It would be helpful to know how many people typically pursue obgyn residency, for example. Did you capture most of them?
  - How many options were students given for priorities for residency matching. It's unclear if "top 3" is meaningful if number of choices is not indicated.
- **Discussion**
  - Consider using percentages instead of “fourth-fiths”
  - Consider adding a statement acknowledging that not all people who can bear children/need abortion care identify as women and there are limitations in this data to represent this group (PMCID: PMC7518170).
  - Please expand on limitations.

Have any limitations of the research been acknowledged?

Partly

Is the work clearly and accurately presented and does it cite the current literature?

Partly

If applicable, is the statistical analysis and its interpretation appropriate?

No

Are all the source data underlying the results available to ensure full reproducibility?

Partly

Is the study design appropriate and is the work technically sound?

Partly

Are the conclusions drawn adequately supported by the results?

Partly

Are sufficient details of methods and analysis provided to allow replication by others?

No

Reviewer Expertise:

medical education, abortion education

We confirm that we have read this submission and believe that we have an appropriate level of expertise to state that we do not consider it to be of an acceptable scientific standard, for reasons outlined above.

# Response

**Published:** 2025-04-25

**Authors:** Zeb Saeed

**Affiliations:** Indiana University Health Inc, Indianapolis, Indiana, USA

Dear Editorial team, We appreciate the detailed and thoughtful comments and recommendations provided by the three reviewers. Please find attached below our responses to each individual comment and changes made in the revised manuscript.   Thank you, Alissa Conklin on behalf of the authors.  

 Reviewer Comment Response Location Reviewer 1    

 This is too long and also does not have a concise logic between the background and the study. Let me try to make some recommendations. In paragraph 1, you don’t need the first two sentences… You can start with Dobbs. We thank you for your comment, and we appreciate you offering ways to make the manuscript more concise. Concerning the first two sentences, we believe that they set the tone for the historical implications of the Dobbs decision. SInce this is an educational journal, we find that highlighting historical elements helps to contextualize problems for the reader. We updated the first sentence to say “historically” rather than “always” to highlight the relevance. Introduction, paragraph 1 The Dobbs sentence can be shorter. I had a hard time following your logic in the next few sentences. You make a big leap from Dobbs not conferring a right to abortion to recommendations for medical education. You should clarify that. Why should we care about UME inclusion of abortion?  It is common, etc. And because ACOG and APGO recommend it. Also, I don’t think APGO went “one step further;” they simply clarified learning objectives for medical students and that they should learn it regardless of personal belief. We shortened the Dobbs sentence to make it more concise. To address the leap from the implications of Dobbs to medical education, we added a statement about how states now have the power to decide whether abortion healthcare was legal.   We removed the “one step further” comment. We also make a brief comment about the importance of privacy and abortion healthcare for women’s health. Introduction, paragraph 2  I don’t understand:  Conflicting priorities between law and associations are at the center of reproductive healthcare education…. I think what you are saying is that the laws causing restrictions in some states will make it hard for medical schools to ensure that medical students have access to clinical exposure to abortion care.  I don’t think the conflicting priorities are at the “center.”  There are many medical schools that – despite being in a restrictive state – have integrated education. Your points are well taken. We have updated this portion of the paragraph to specify how this problem is specific to states with restrictions and we deleted the comment about these tensions being at the “center” of this issue. Introduction, paragraph 1 In paragraph 2 you make an important point but it is also written in a way that is confusing.  You could be more straightforward.  In addition to restrictive laws limiting clinical abortion care, the laws will affect students’ access to comprehensive health care. We updated the paragraph to be more straightforward, and used your recommendation as a way to make our point more clear. Introduction, paragraph 2 Paragraph 3 should also be shortened.  I like that you review how many medical students are training in restrictive states.  You should introduce Indiana later - in paragraph 5. It’s not clear why you are mentioning it until you are making the argument for a study in Indiana. We have shortened the paragraph and moved elements pertaining to Indiana to paragraph 5. Introduction, paragraph 3 Paragraph 4 should be integrated with paragraph 2. We agree and have integrated the two paragraphs into one. Introduction, paragraph 2 You should integrate the Indiana data in paragraph 5. And I don’t think you need to make the case that the media coverage and Caitlin’s case makes us want to study Indiana. I think it’s just as important that it has a lot of medical students and it’s restrictive… The data could help us understand the experiences of medical students elsewhere.   We agree. We’ve removed the comment about Dr. Bernard and how the national spotlight has created implications for this work. Introduction, paragraph 5 I like that you first frame the importance of medical student education, then you talk about the fact that students are often of reproductive age and that they are patients who may need access to abortion thus it affects their health care access (combine the two paragraphs), then you talk about how many students are training in states with restrictions and link the two concepts – lack of training and also concern that they will not stay there or will leave limiting access for patients, then the link to the study. Thank you for providing this summary. We believe we have incorporated your feedback, and we find that your suggestions enhance the flow of the manuscript. Introduction how did you design the survey? Did you pilot it? Did you use questions from other surveys?  I like the reflexivity paragraph but would recommend moving it down. And it should be shorter. And how did your positionality affect the study? Thank you for your thoughtful questions. Regarding your questions about the design of the survey: we have included this paragraph in our methods: “The survey was designed by all three authors together through an iterative process with multiple editions before finalizing the survey. At the time of its development in 2022, there were no validated instruments or similar surveys to emulate. Hence, we modeled several of our questions after the only publicly available survey at the time, a BestColleges' survey of undergraduate and graduate students. To identify relevant factors that may influence residency choices for students, we sought guidance from the 2021 NRMP Applicant Survey, in particular, on data on applicants pursuing OB/GYN residency. To ensure the timeliness of this survey in the midst of changing laws, we decided to forego piloting the survey instead of delaying it.”     We appreciate your liking our reflexivity paragraph. As suggested, we have moved it down and added how we mitigated bias while being conscious of our positionality. Methods, paragraphs 1 and 4 I like the idea that you had a framework for analysis, but I’m confused by its application to this quantitative, cross-sectional survey.  I don’t see evidence that you used it to design the study or the survey. The questions don’t make links to structural impacts on inequities or really any structural interpretation or insights. You ask only about demographics, and looking at differences by sex, gender, and other demographic factors does not necessarily reflect structural effects on inequities.  It is a big jump for me that you focused on rearticulating cultural presentations in structural terms based on such a simple cross-sectional quantitative survey. Perhaps you did ask questions that reflect structural competency and if so please describe. If what I understand is correct – that you did not use the framework in designing the study and survey, I would instead present the data and then apply some of these concepts as possible interpretations of the data. What you are getting at is potential structural impacts of the findings. Thank you for the thoughtful comments. We recognize and agree that our study's cross-sectional survey-based design limits direct measurement of structural forces. We have revised the manuscript to clarify that we used structural competency as an interpretive lens rather than a design framework, to focus on identities which would be impacted the most from abortion restrictions as a structural factor.    Methods Paragraph 3     How did you ask about sex, gender, religion, race, ethnicity, etc?  Was Hispanic ethnicity asked as a separate question from race? Also, I don’t think you asked about religious importance, only religious identity. Thank you for asking these very important questions. Please note that we uploaded the actual survey for review which is publicly available here. As you may see, we tried to be inclusive when asking for gender identity and allowed a space for individuals to write if their gender was not amongst the chosen ones. We used the same approach for religion. In terms of race and ethnicity, while both were asked in one question, individuals could and did identify having more than one race and/or ethnicity. We do recognize that we should have ideally asked about ethnicity separately from race as this is an opportunity of growth for us. Regarding religious importance: You are right, that is an important distinction and for brevity we did not include that question which is a limitation to our study and has been added as such. Discussion, paragraph 8 “Only 18 students voluntarily disclosed information about past abortions” – this is a very biased sentence.  It sounds like you assume more have had abortions.  The same could be true about how they reported their past pregnancies, or how many had children. I would just present the data. Thank you so much for pointing this out. We agree and have reworded that sentence and removed the word Only. Results, paragraph 2 Why did you choose sex instead of gender? From a sociocultural and structural perspective, gender is more important. I’m assuming you asked because you were curious about differences by having the reproductive potential for pregnancy? I would make that clear. Also as I asked above how did you ask about sex and gender? Which is presented in the first paragraph of Results when you say “female”? Thank you for your comments. We asked about both gender and sex. As you pointed out, we wanted to explore differences in perceptions and beliefs among those having reproductive potential for pregnancy as the Dobbs decision would directly impact them. Please refer to previous comment on how gender and sex were asked. We would also like to point out that we did do analysis among different genders and had identical results i.e among those individuals who identified as women. Results, under section “ Demographic influences on personal beliefs and plans after the Dobbs decision”   This section is too long. You don’t need to report all the details in sentences since you have nice tables. We appreciate your suggestion and have done so. Results I would significantly simplify your discussion. We appreciate this suggestion and have removed several areas that were either repetitive or suggestive to simplify our discussion. Discussion I am confused about the relationship between the survey results to how you are seeing structural competency.  For example, I do not think you “focus on rearticulating the cultural presentation of abortion”. You analyze the data by sex and other demographics. You found that some people with the potential to become pregnant and/or are socialized as women make different decisions and have different opinions than those without pregnancy potential and/or are socialized as man, and this may be based on protecting their reproductive autonomy and access to miscarriage and abortion care. Career decision making includes personal and other factors.  I’m also not seeing the connection with structures that support health. Thank you for this perspective.  We agree the relationship between the survey results and structural competency was unclear.  We have modified this accordingly. Discussion, paragraph 1 and 2 I don’t have time to go through every paragraph, but I recommend that you focus this on the implication for medical education and the workforce.  What other studies look at career decision making.  You say “this is one of the first studies designed to collect demographic data and personal views.” Perhaps you should be more specific. Many studies about integration of abortion care in practice have included personal beliefs about abortion. What are the potential implications on health care inequities? We agree with your recommendation to be more specific and focus more on health inequities and these modifications have been made. Discussion, paragraph 5 and 6 I don’t think it is a strength that you all work at IU. I do think it is a strength that you surveyed students at a large medical school in a restrictive state. Thank you for this suggestion.  We have modified accordingly. Discussion,paragraph 7 I would significantly shorten all parts of the discussion. For example, you could shorten the paragraph on religion. I think you are stretching the implications – for example the last two sentences don’t really connect to the data, and I’m not sure how they relate to each other. Are you trying to say that the religious demographics of the US is proof of long lasting impacts? We have shortened the discussion throughout.  We have also simplified the discussion on religion. We have also modified the connection regarding religious demographics. Discussion, paragraph 5 your table titles should stand alone so that someone can just look at them and know what the study is about. So they should be something like: Demographics of IU medical students responding to a survey about abortion attitudes and career plans… In table 1 what does “Having children” mean? Thank you for your suggestion. We have modified the table heading per your suggestion. Having children meant currently being a biological, foster or adopted parent Table 1 Reviewer 2     Thank you for the opportunity to review this manuscript, which departs from the Dobbs decision on federal restrictive abortion laws to examine its implications for maternal mortality rates and the medical field. This study aims to analyze the perceptions of third- and fourth-year medical students from one state university regarding how the state's current abortion restrictions may impact their career choices.   The abstract is concise and clear. Overall, the manuscript is written in a somewhat colloquial and practical manner, making it easy to read. We thank you for reviewing our manuscript and providing feedback. We were especially intentional about making the manuscript colloquial and easy to read, so we appreciate you saying we have achieved our goal. Thank you again.   The authors describe their methodology, which centers on an anonymous survey conducted at Indiana University School of Medicine between December 2022 and March 2023. The survey included questions about personal beliefs on abortion, current abortion laws in Indiana, and factors influencing the choice of residency training and practice locations. However, we wonder about the process used to establish the reliability and validity of the measurement instrument. We suggest conducting a mixed-method investigation to gain a deeper understanding of the perceptions of Indiana medical students in their third and fourth years of education following the Dobbs decision. This could help assess whether the state’s current abortion restrictions impact their choices regarding residency training and where they ultimately wish to practice and live. An explanation of this process would enrich the manuscript. Thank you for your comments. We agree that in an ideal scenario, we would have either conducted a pilot survey or used a validated instrument for this study. However, to ensure the timeliness of this survey in the midst of changing laws, we decided to forego piloting the survey instead of delaying it.  At the time of the survey development in 2022, there were no validated instruments or similar surveys to emulate. Hence, we modeled several of our questions after the only publicly available survey at the time, a BestColleges' survey of undergraduate and graduate students. To identify relevant factors that may influence residency choices for students, we sought guidance from the 2021 NRMP Applicant Survey, in particular, on data on applicants pursuing OB/GYN residency. This has been added to the methods section of the manuscript. We appreciate the suggestion of having a mixed-methods study design and while this was outside of scope for this study; we would certainly consider this for future research. Methods, paragraph 1 Despite the limitations of a single-center investigation and the strategically selected population in a "more conservative state," the sample size is significant. We appreciate your comments. As you pointed out, Indiana is a more conservative state with the largest medical school in the US, hence we consider our study to be an important addition to the medical education literature   The tables and diagrams guide the reader through the manuscript. The survey results indicate that four-fifths of Indiana medical students disagreed with the Dobbs decision. Initially, 71.4% of students had not considered state abortion laws when selecting a medical school. However, after the Dobbs decision, 66.3% of third-year and 40.3% of fourth-year students indicated they would now consider abortion laws when choosing a residency program. Additionally, 47.5% of female students stated they would seek residency in a state where abortion is legal, and 55.3% of single students were more likely to leave Indiana to practice medicine. Thank you for your kind comments and they are appreciated.   The discussion section includes both strengths and limitations of the research, suggesting that physicians with more liberal views on abortion are less likely to work in conservative states. The manuscript examines how socio-cultural environments can shape medical education, career choices, and practice locations. The authors warn that this trend may compromise patient care and emphasize the importance of abortion laws in shaping the healthcare workforce and influencing women's reproductive decisions and medical careers. Thank you for your kind comments and they are appreciated.   The manuscript aims to present a specific viewpoint and highlights associations worth reflecting on, opening a potential avenue for further investigation. This perspective is likely to capture the attention of the health professions education community and stimulate conversation by raising similar or dissenting opinions. While reading this manuscript, one might recall Freire’s statement: “There is no such thing as a neutral education process” (1). This notion prompts reflection on education as either a tool for freedom or, conversely, a means of conforming to and reinforcing the *status quo*.   We believe we highlighted the reinforcement of the status quo in our Discussion paragraph regarding religion (paragraph 5) and also reviews the challenges facing the health professions education community on this topic. Discussion, paragraph 5 In conclusion, we believe that, despite the need for a more thorough articulation of the study's limitations, the manuscript holds inherent value and is worth reading. It may potentially inspire others to advance evidence-based perspectives in this field. Perhaps the manuscript would better fit the format of a perspective piece. We appreciate this recommendation and have expanded the limitations. Discussion, paragraph 8 Reviewer 3     This is an important topic and the authors clearly justify why medical students in Indiana are a good specific test scenario (largest med school, restrictive laws, maternal mortality rates).   However, we have some concerns about the low response rate (23%) which makes it difficult to draw conclusions about impact on medical student careers. More details on non-responders/the entire 3rd/4th year demographics would be helpful in clarifying if this is a representative sample. The authors do say this in their discussion but do not give specific data to clearly support this.   Additionally there is significant information missing in the methods section including outcomes of interest, how missing data was approached, and what answer choices were given as options (e.g. in priorities for residency match, the authors do not specify how many options were given before saying 19% chose abortion laws in their top 3).Recommend referring to STROBE checklist to make sure all methodology is included in report. (https://www.strobe-statement.org/checklists/). The conclusions must be adapted to the limitations of the study. In this current version, these conclusions are too strong/certain for what is presented. Thank you for your thoughtful comments and feedback. We have provided answers to each comment in the following rows.   For background, clarify what "the impact of this ruling may be huge on the medical field" -link to this topic specifically, as in "it is unknown what the impact of this ruling is on medical student career choices in Indiana"   We have changed this sentence to state the ruling “may have significant impact on healthcare in the state.” Abstract: Background Results: Add 23% response rate to Results section as first sentence. then say, of the students who responded... Thank you for your comment. We are not sure what you may be referring to as we do specify in the first section that that 178 of 763 students started the survey so 23.3% responded to it. Results, paragraph 1 Use percents instead of fractions (e.g. four-fifths) Thank you and we have done so as appropriate. Discussion, paragraph 1 Conclusion: Be careful about using terms like liberal in regards to abortion views. Be more specific on what you can state based on the questions you asked We appreciate this and have adjusted accordingly. Conclusion Consider removing “went one step further” in the following sentence: In addition, the Association of Professors of Gynecology and Obstetrics (APGO) went one step further and asserted, “Regardless of personal views about abortion, students should be knowledgeable about its public health importance, as well as techniques and patient safety implications” 5. Thank you for this comment. We have removed the phrase, “went one step further.” Introduction, paragraph 2 Consider changing abortion to abortion care where applicable throughout the manuscript.   We have changed abortion to abortion healthcare where applicable. Throughout Unclear how reference 15 is being used where it is cited. Please provide further clarification. We have removed that reference and the statements preceding it. Introduction, paragraph 4 Consider rephrasing “In the case of Dr. Caitlin Bernard, the nation has witnessed how a physician can be scrutinized and reprimanded for doing their job, and how abortion law restrictions and political motivations can disrupt work and life 16. “ → from “doing their job” to “providing abortion care”. Also not every reader will be familiar with this case, therefore it may be reasonable to provide further clarification. We have removed this section based on feedback from reviewer 1. The feedback suggested it was unnecessary to communicate the importance of this study. Introduction Decide on wording women or females and be consistent. Prefer women as female is used as a descriptor for animals (e.g. female mouse) We have updated the manuscript to have consistent “woman” language. Throughout the manuscript. an outcome is not specified Thank you for your comment. The outcome is the response to the survey questions which aimed at asking students’ perceptions. This has been clarified in the methods. Introduction and Methods Please refer to the STROBE checklist to make sure you're including all important information including: variables, methods of assessment, and statistical methods for data analysis. How was missing data analyzed for the students who started but did not complete entire survey? Were there responses included?   Thank you for your comments. We have modified the methods to include all the request information in accordance with the STROBE checklist. We did not include responses of the 10 students who started but did not complete the entire survey and this has been now clarified in the results. Results, paragraph 1 It would be helpful to compare respondents demographics to non-respondent demographics (or to the whole 3rd and 4th year demographics) to show that it is in fact representative (adapt Table 1 to include a second column representing the entire 3rd/4th year class with as much information as you have. Thank you for your comment and suggestion.  Though we cannot collect demographic information of students who didn’t respond as would be outside of the scope of our IRB, the distribution of sex and race in our cohort mirror publicly available data on IU school of medicine student body.           Results, paragraph 1 It would be helpful to know how many people typically pursue obgyn residency, for example. Did you capture most of them? Typically, anywhere from 10 to 30 students each year pursue OB-GYN residency. However, it would unclear if we captured all or most of them as the abortion laws may have changed this number significantly.   How many options were students given for priorities for residency matching. It's unclear if "top 3" is meaningful if number of choices is not indicated. Thank you for your question. While this information is available in the appendix to the paper, we also added in the results that students could choose between six different factors. Results, paragraph 4 Consider using percentages instead of “fourth-fiths”   This was modified. Discussion, paragraph 1 Consider adding a statement acknowledging that not all people who can bear children/need abortion care identify as women and there are limitations in this data to represent this group (PMCID: PMC7518170). This is an astute observation, and our manuscript has been modified.  Thank you for the reference, this has also been added. Discussion, paragraph 8 Please expand on limitations. Thank you for your suggestion. It is duly noted and done. Discussion, paragraph 8     Dear Editorial team, We appreciate the detailed and thoughtful comments and recommendations provided by the three reviewers. Please find attached below our responses to each individual comment and changes made in the revised manuscript.   Thank you, Alissa Conklin on behalf of the authors.   Reviewer Comment Response Location Reviewer 1     This is too long and also does not have a concise logic between the background and the study. Let me try to make some recommendations. In paragraph 1, you don’t need the first two sentences… You can start with Dobbs. We thank you for your comment, and we appreciate you offering ways to make the manuscript more concise. Concerning the first two sentences, we believe that they set the tone for the historical implications of the Dobbs decision. SInce this is an educational journal, we find that highlighting historical elements helps to contextualize problems for the reader. We updated the first sentence to say “historically” rather than “always” to highlight the relevance. Introduction, paragraph 1 The Dobbs sentence can be shorter. I had a hard time following your logic in the next few sentences. You make a big leap from Dobbs not conferring a right to abortion to recommendations for medical education. You should clarify that. Why should we care about UME inclusion of abortion?  It is common, etc. And because ACOG and APGO recommend it. Also, I don’t think APGO went “one step further;” they simply clarified learning objectives for medical students and that they should learn it regardless of personal belief. We shortened the Dobbs sentence to make it more concise. To address the leap from the implications of Dobbs to medical education, we added a statement about how states now have the power to decide whether abortion healthcare was legal.   We removed the “one step further” comment. We also make a brief comment about the importance of privacy and abortion healthcare for women’s health. Introduction, paragraph 2  I don’t understand:  Conflicting priorities between law and associations are at the center of reproductive healthcare education…. I think what you are saying is that the laws causing restrictions in some states will make it hard for medical schools to ensure that medical students have access to clinical exposure to abortion care.  I don’t think the conflicting priorities are at the “center.”  There are many medical schools that – despite being in a restrictive state – have integrated education. Your points are well taken. We have updated this portion of the paragraph to specify how this problem is specific to states with restrictions and we deleted the comment about these tensions being at the “center” of this issue. Introduction, paragraph 1 In paragraph 2 you make an important point but it is also written in a way that is confusing.  You could be more straightforward.  In addition to restrictive laws limiting clinical abortion care, the laws will affect students’ access to comprehensive health care. We updated the paragraph to be more straightforward, and used your recommendation as a way to make our point more clear. Introduction, paragraph 2 Paragraph 3 should also be shortened.  I like that you review how many medical students are training in restrictive states.  You should introduce Indiana later - in paragraph 5. It’s not clear why you are mentioning it until you are making the argument for a study in Indiana. We have shortened the paragraph and moved elements pertaining to Indiana to paragraph 5. Introduction, paragraph 3 Paragraph 4 should be integrated with paragraph 2. We agree and have integrated the two paragraphs into one. Introduction, paragraph 2 You should integrate the Indiana data in paragraph 5. And I don’t think you need to make the case that the media coverage and Caitlin’s case makes us want to study Indiana. I think it’s just as important that it has a lot of medical students and it’s restrictive… The data could help us understand the experiences of medical students elsewhere.   We agree. We’ve removed the comment about Dr. Bernard and how the national spotlight has created implications for this work. Introduction, paragraph 5 I like that you first frame the importance of medical student education, then you talk about the fact that students are often of reproductive age and that they are patients who may need access to abortion thus it affects their health care access (combine the two paragraphs), then you talk about how many students are training in states with restrictions and link the two concepts – lack of training and also concern that they will not stay there or will leave limiting access for patients, then the link to the study. Thank you for providing this summary. We believe we have incorporated your feedback, and we find that your suggestions enhance the flow of the manuscript. Introduction how did you design the survey? Did you pilot it? Did you use questions from other surveys?  I like the reflexivity paragraph but would recommend moving it down. And it should be shorter. And how did your positionality affect the study? Thank you for your thoughtful questions. Regarding your questions about the design of the survey: we have included this paragraph in our methods: “The survey was designed by all three authors together through an iterative process with multiple editions before finalizing the survey. At the time of its development in 2022, there were no validated instruments or similar surveys to emulate. Hence, we modeled several of our questions after the only publicly available survey at the time, a BestColleges' survey of undergraduate and graduate students. To identify relevant factors that may influence residency choices for students, we sought guidance from the 2021 NRMP Applicant Survey, in particular, on data on applicants pursuing OB/GYN residency. To ensure the timeliness of this survey in the midst of changing laws, we decided to forego piloting the survey instead of delaying it.”     We appreciate your liking our reflexivity paragraph. As suggested, we have moved it down and added how we mitigated bias while being conscious of our positionality. Methods, paragraphs 1 and 4 I like the idea that you had a framework for analysis, but I’m confused by its application to this quantitative, cross-sectional survey.  I don’t see evidence that you used it to design the study or the survey. The questions don’t make links to structural impacts on inequities or really any structural interpretation or insights. You ask only about demographics, and looking at differences by sex, gender, and other demographic factors does not necessarily reflect structural effects on inequities.  It is a big jump for me that you focused on rearticulating cultural presentations in structural terms based on such a simple cross-sectional quantitative survey. Perhaps you did ask questions that reflect structural competency and if so please describe. If what I understand is correct – that you did not use the framework in designing the study and survey, I would instead present the data and then apply some of these concepts as possible interpretations of the data. What you are getting at is potential structural impacts of the findings. Thank you for the thoughtful comments. We recognize and agree that our study's cross-sectional survey-based design limits direct measurement of structural forces. We have revised the manuscript to clarify that we used structural competency as an interpretive lens rather than a design framework, to focus on identities which would be impacted the most from abortion restrictions as a structural factor.    Methods Paragraph 3     How did you ask about sex, gender, religion, race, ethnicity, etc?  Was Hispanic ethnicity asked as a separate question from race? Also, I don’t think you asked about religious importance, only religious identity. Thank you for asking these very important questions. Please note that we uploaded the actual survey for review which is publicly available here. As you may see, we tried to be inclusive when asking for gender identity and allowed a space for individuals to write if their gender was not amongst the chosen ones. We used the same approach for religion. In terms of race and ethnicity, while both were asked in one question, individuals could and did identify having more than one race and/or ethnicity. We do recognize that we should have ideally asked about ethnicity separately from race as this is an opportunity of growth for us. Regarding religious importance: You are right, that is an important distinction and for brevity we did not include that question which is a limitation to our study and has been added as such. Discussion, paragraph 8 “Only 18 students voluntarily disclosed information about past abortions” – this is a very biased sentence.  It sounds like you assume more have had abortions.  The same could be true about how they reported their past pregnancies, or how many had children. I would just present the data. Thank you so much for pointing this out. We agree and have reworded that sentence and removed the word Only. Results, paragraph 2 Why did you choose sex instead of gender? From a sociocultural and structural perspective, gender is more important. I’m assuming you asked because you were curious about differences by having the reproductive potential for pregnancy? I would make that clear. Also as I asked above how did you ask about sex and gender? Which is presented in the first paragraph of Results when you say “female”? Thank you for your comments. We asked about both gender and sex. As you pointed out, we wanted to explore differences in perceptions and beliefs among those having reproductive potential for pregnancy as the Dobbs decision would directly impact them. Please refer to previous comment on how gender and sex were asked. We would also like to point out that we did do analysis among different genders and had identical results i.e among those individuals who identified as women. Results, under section “ Demographic influences on personal beliefs and plans after the Dobbs decision”   This section is too long. You don’t need to report all the details in sentences since you have nice tables. We appreciate your suggestion and have done so. Results I would significantly simplify your discussion. We appreciate this suggestion and have removed several areas that were either repetitive or suggestive to simplify our discussion. Discussion I am confused about the relationship between the survey results to how you are seeing structural competency.  For example, I do not think you “focus on rearticulating the cultural presentation of abortion”. You analyze the data by sex and other demographics. You found that some people with the potential to become pregnant and/or are socialized as women make different decisions and have different opinions than those without pregnancy potential and/or are socialized as man, and this may be based on protecting their reproductive autonomy and access to miscarriage and abortion care. Career decision making includes personal and other factors.  I’m also not seeing the connection with structures that support health. Thank you for this perspective.  We agree the relationship between the survey results and structural competency was unclear.  We have modified this accordingly. Discussion, paragraph 1 and 2 I don’t have time to go through every paragraph, but I recommend that you focus this on the implication for medical education and the workforce.  What other studies look at career decision making.  You say “this is one of the first studies designed to collect demographic data and personal views.” Perhaps you should be more specific. Many studies about integration of abortion care in practice have included personal beliefs about abortion. What are the potential implications on health care inequities? We agree with your recommendation to be more specific and focus more on health inequities and these modifications have been made. Discussion, paragraph 5 and 6 I don’t think it is a strength that you all work at IU. I do think it is a strength that you surveyed students at a large medical school in a restrictive state. Thank you for this suggestion.  We have modified accordingly. Discussion,paragraph 7 I would significantly shorten all parts of the discussion. For example, you could shorten the paragraph on religion. I think you are stretching the implications – for example the last two sentences don’t really connect to the data, and I’m not sure how they relate to each other. Are you trying to say that the religious demographics of the US is proof of long lasting impacts? We have shortened the discussion throughout.  We have also simplified the discussion on religion. We have also modified the connection regarding religious demographics. Discussion, paragraph 5 your table titles should stand alone so that someone can just look at them and know what the study is about. So they should be something like: Demographics of IU medical students responding to a survey about abortion attitudes and career plans… In table 1 what does “Having children” mean? Thank you for your suggestion. We have modified the table heading per your suggestion. Having children meant currently being a biological, foster or adopted parent Table 1 Reviewer 2     Thank you for the opportunity to review this manuscript, which departs from the Dobbs decision on federal restrictive abortion laws to examine its implications for maternal mortality rates and the medical field. This study aims to analyze the perceptions of third- and fourth-year medical students from one state university regarding how the state's current abortion restrictions may impact their career choices.   The abstract is concise and clear. Overall, the manuscript is written in a somewhat colloquial and practical manner, making it easy to read. We thank you for reviewing our manuscript and providing feedback. We were especially intentional about making the manuscript colloquial and easy to read, so we appreciate you saying we have achieved our goal. Thank you again.   The authors describe their methodology, which centers on an anonymous survey conducted at Indiana University School of Medicine between December 2022 and March 2023. The survey included questions about personal beliefs on abortion, current abortion laws in Indiana, and factors influencing the choice of residency training and practice locations. However, we wonder about the process used to establish the reliability and validity of the measurement instrument. We suggest conducting a mixed-method investigation to gain a deeper understanding of the perceptions of Indiana medical students in their third and fourth years of education following the Dobbs decision. This could help assess whether the state’s current abortion restrictions impact their choices regarding residency training and where they ultimately wish to practice and live. An explanation of this process would enrich the manuscript. Thank you for your comments. We agree that in an ideal scenario, we would have either conducted a pilot survey or used a validated instrument for this study. However, to ensure the timeliness of this survey in the midst of changing laws, we decided to forego piloting the survey instead of delaying it.  At the time of the survey development in 2022, there were no validated instruments or similar surveys to emulate. Hence, we modeled several of our questions after the only publicly available survey at the time, a BestColleges' survey of undergraduate and graduate students. To identify relevant factors that may influence residency choices for students, we sought guidance from the 2021 NRMP Applicant Survey, in particular, on data on applicants pursuing OB/GYN residency. This has been added to the methods section of the manuscript. We appreciate the suggestion of having a mixed-methods study design and while this was outside of scope for this study; we would certainly consider this for future research. Methods, paragraph 1 Despite the limitations of a single-center investigation and the strategically selected population in a "more conservative state," the sample size is significant. We appreciate your comments. As you pointed out, Indiana is a more conservative state with the largest medical school in the US, hence we consider our study to be an important addition to the medical education literature   The tables and diagrams guide the reader through the manuscript. The survey results indicate that four-fifths of Indiana medical students disagreed with the Dobbs decision. Initially, 71.4% of students had not considered state abortion laws when selecting a medical school. However, after the Dobbs decision, 66.3% of third-year and 40.3% of fourth-year students indicated they would now consider abortion laws when choosing a residency program. Additionally, 47.5% of female students stated they would seek residency in a state where abortion is legal, and 55.3% of single students were more likely to leave Indiana to practice medicine. Thank you for your kind comments and they are appreciated.   The discussion section includes both strengths and limitations of the research, suggesting that physicians with more liberal views on abortion are less likely to work in conservative states. The manuscript examines how socio-cultural environments can shape medical education, career choices, and practice locations. The authors warn that this trend may compromise patient care and emphasize the importance of abortion laws in shaping the healthcare workforce and influencing women's reproductive decisions and medical careers. Thank you for your kind comments and they are appreciated.   The manuscript aims to present a specific viewpoint and highlights associations worth reflecting on, opening a potential avenue for further investigation. This perspective is likely to capture the attention of the health professions education community and stimulate conversation by raising similar or dissenting opinions. While reading this manuscript, one might recall Freire’s statement: “There is no such thing as a neutral education process” (1). This notion prompts reflection on education as either a tool for freedom or, conversely, a means of conforming to and reinforcing the *status quo*.   We believe we highlighted the reinforcement of the status quo in our Discussion paragraph regarding religion (paragraph 5) and also reviews the challenges facing the health professions education community on this topic. Discussion, paragraph 5 In conclusion, we believe that, despite the need for a more thorough articulation of the study's limitations, the manuscript holds inherent value and is worth reading. It may potentially inspire others to advance evidence-based perspectives in this field. Perhaps the manuscript would better fit the format of a perspective piece. We appreciate this recommendation and have expanded the limitations. Discussion, paragraph 8 Reviewer 3     This is an important topic and the authors clearly justify why medical students in Indiana are a good specific test scenario (largest med school, restrictive laws, maternal mortality rates).   However, we have some concerns about the low response rate (23%) which makes it difficult to draw conclusions about impact on medical student careers. More details on non-responders/the entire 3rd/4th year demographics would be helpful in clarifying if this is a representative sample. The authors do say this in their discussion but do not give specific data to clearly support this.   Additionally there is significant information missing in the methods section including outcomes of interest, how missing data was approached, and what answer choices were given as options (e.g. in priorities for residency match, the authors do not specify how many options were given before saying 19% chose abortion laws in their top 3).Recommend referring to STROBE checklist to make sure all methodology is included in report. (https://www.strobe-statement.org/checklists/). The conclusions must be adapted to the limitations of the study. In this current version, these conclusions are too strong/certain for what is presented. Thank you for your thoughtful comments and feedback. We have provided answers to each comment in the following rows.   For background, clarify what "the impact of this ruling may be huge on the medical field" -link to this topic specifically, as in "it is unknown what the impact of this ruling is on medical student career choices in Indiana"   We have changed this sentence to state the ruling “may have significant impact on healthcare in the state.” Abstract: Background Results: Add 23% response rate to Results section as first sentence. then say, of the students who responded... Thank you for your comment. We are not sure what you may be referring to as we do specify in the first section that that 178 of 763 students started the survey so 23.3% responded to it. Results, paragraph 1 Use percents instead of fractions (e.g. four-fifths) Thank you and we have done so as appropriate. Discussion, paragraph 1 Conclusion: Be careful about using terms like liberal in regards to abortion views. Be more specific on what you can state based on the questions you asked We appreciate this and have adjusted accordingly. Conclusion Consider removing “went one step further” in the following sentence: In addition, the Association of Professors of Gynecology and Obstetrics (APGO) went one step further and asserted, “Regardless of personal views about abortion, students should be knowledgeable about its public health importance, as well as techniques and patient safety implications” 5. Thank you for this comment. We have removed the phrase, “went one step further.” Introduction, paragraph 2 Consider changing abortion to abortion care where applicable throughout the manuscript.   We have changed abortion to abortion healthcare where applicable. Throughout Unclear how reference 15 is being used where it is cited. Please provide further clarification. We have removed that reference and the statements preceding it. Introduction, paragraph 4 Consider rephrasing “In the case of Dr. Caitlin Bernard, the nation has witnessed how a physician can be scrutinized and reprimanded for doing their job, and how abortion law restrictions and political motivations can disrupt work and life 16. “ → from “doing their job” to “providing abortion care”. Also not every reader will be familiar with this case, therefore it may be reasonable to provide further clarification. We have removed this section based on feedback from reviewer 1. The feedback suggested it was unnecessary to communicate the importance of this study. Introduction Decide on wording women or females and be consistent. Prefer women as female is used as a descriptor for animals (e.g. female mouse) We have updated the manuscript to have consistent “woman” language. Throughout the manuscript. an outcome is not specified Thank you for your comment. The outcome is the response to the survey questions which aimed at asking students’ perceptions. This has been clarified in the methods. Introduction and Methods Please refer to the STROBE checklist to make sure you're including all important information including: variables, methods of assessment, and statistical methods for data analysis. How was missing data analyzed for the students who started but did not complete entire survey? Were there responses included?   Thank you for your comments. We have modified the methods to include all the request information in accordance with the STROBE checklist. We did not include responses of the 10 students who started but did not complete the entire survey and this has been now clarified in the results. Results, paragraph 1 It would be helpful to compare respondents demographics to non-respondent demographics (or to the whole 3rd and 4th year demographics) to show that it is in fact representative (adapt Table 1 to include a second column representing the entire 3rd/4th year class with as much information as you have. Thank you for your comment and suggestion.  Though we cannot collect demographic information of students who didn’t respond as would be outside of the scope of our IRB, the distribution of sex and race in our cohort mirror publicly available data on IU school of medicine student body.           Results, paragraph 1 It would be helpful to know how many people typically pursue obgyn residency, for example. Did you capture most of them? Typically, anywhere from 10 to 30 students each year pursue OB-GYN residency. However, it would unclear if we captured all or most of them as the abortion laws may have changed this number significantly.   How many options were students given for priorities for residency matching. It's unclear if "top 3" is meaningful if number of choices is not indicated. Thank you for your question. While this information is available in the appendix to the paper, we also added in the results that students could choose between six different factors. Results, paragraph 4 Consider using percentages instead of “fourth-fiths”   This was modified. Discussion, paragraph 1 Consider adding a statement acknowledging that not all people who can bear children/need abortion care identify as women and there are limitations in this data to represent this group (PMCID: PMC7518170). This is an astute observation, and our manuscript has been modified.  Thank you for the reference, this has also been added. Discussion, paragraph 8 Please expand on limitations. Thank you for your suggestion. It is duly noted and done. Discussion, paragraph 8

# Response

**Published:** 2025-05-04

**Authors:** Zeb Saeed

**Affiliations:** Indiana University Health Inc, Indianapolis, Indiana, USA

Response to reviewer 1

1. This is too long and also does not have a concise logic between the background and the study. Let me try to make some recommendations.

In paragraph 1, you don’t need the first two sentences… You can start with Dobbs. Response: We thank you for your comment, and we appreciate you offering ways to make the manuscript more concise. Concerning the first two sentences, we believe that they set the tone for the historical implications of the Dobbs decision. SInce this is an educational journal, we find that highlighting historical elements helps to contextualize problems for the reader. We updated the first sentence to say “historically” rather than “always” to highlight the relevance.

1. The Dobbs sentence can be shorter. I had a hard time following your logic in the next few sentences. You make a big leap from Dobbs not conferring a right to abortion to recommendations for medical education. You should clarify that. Why should we care about UME inclusion of abortion?  It is common, etc. And because ACOG and APGO recommend it. Also, I don’t think APGO went “one step further;” they simply clarified learning objectives for medical students and that they should learn it regardless of personal belief.

Response: We shortened the Dobbs sentence to make it more concise. To address the leap from the implications of Dobbs to medical education, we added a statement about how states now have the power to decide whether abortion healthcare was legal. We removed the “one step further” comment. We also make a brief comment about the importance of privacy and abortion healthcare for women’s health.  

1. I don’t understand:  Conflicting priorities between law and associations are at the center of reproductive healthcare education…. I think what you are saying is that the laws causing restrictions in some states will make it hard for medical schools to ensure that medical students have access to clinical exposure to abortion care.  I don’t think the conflicting priorities are at the “center.”  There are many medical schools that – despite being in a restrictive state – have integrated education.

Response: Your points are well taken. We have updated this portion of the paragraph to specify how this problem is specific to states with restrictions and we deleted the comment about these tensions being at the “center” of this issue.  

1. In paragraph 2 you make an important point but it is also written in a way that is confusing.  You could be more straightforward.  In addition to restrictive laws limiting clinical abortion care, the laws will affect students’ access to comprehensive health care.

Response: We updated the paragraph to be more straightforward and used your recommendation as a way to make our point more clear.  

1. Paragraph 3 should also be shortened.  I like that you review how many medical students are training in restrictive states.  You should introduce Indiana later - in paragraph 5. It’s not clear why you are mentioning it until you are making the argument for a study in Indiana.Response: We have shortened the paragraph and moved elements pertaining to Indiana to paragraph 5.
2. Paragraph 4 should be integrated with paragraph 2.

Response: We agree and have integrated the two paragraphs into one.

1. You should integrate the Indiana data in paragraph 5. And I don’t think you need to make the case that the media coverage and Caitlin’s case makes us want to study Indiana. I think it’s just as important that it has a lot of medical students and it’s restrictive… The data could help us understand the experiences of medical students elsewhere.

Response: We agree. We’ve removed the comment about Dr. Bernard and how the national spotlight has created implications for this work.

1. I like that you first frame the importance of medical student education, then you talk about the fact that students are often of reproductive age and that they are patients who may need access to abortion thus it affects their health care access (combine the two paragraphs), then you talk about how many students are training in states with restrictions and link the two concepts – lack of training and also concern that they will not stay there or will leave limiting access for patients, then the link to the study.

Response: Thank you for providing this summary. We believe we have incorporated your feedback, and we find that your suggestions enhance the flow of the manuscript how did you design the survey?  Did you pilot it? Did you use questions from other surveys? 

1. I like the reflexivity paragraph but would recommend moving it down. And it should be shorter. And how did your positionality affect the study?

Response: Thank you for your thoughtful questions. Regarding your questions about the design of the survey: we have included this paragraph in our methods: “The survey was designed by all three authors together through an iterative process with multiple editions before finalizing the survey. At the time of its development in 2022, there were no validated instruments or similar surveys to emulate. Hence, we modeled several of our questions after the only publicly available survey at the time, a BestColleges' survey of undergraduate and graduate students. To identify relevant factors that may influence residency choices for students, we sought guidance from the 2021 NRMP Applicant Survey, in particular, on data on applicants pursuing OB/GYN residency. To ensure the timeliness of this survey in the midst of changing laws, we decided to forego piloting the survey instead of delaying it.” We appreciate your liking our reflexivity paragraph. As suggested, we have moved it down and added how we mitigated bias while being conscious of our positionality.  

1. I like the idea that you had a framework for analysis, but I’m confused by its application to this quantitative, cross-sectional survey.  I don’t see evidence that you used it to design the study or the survey. The questions don’t make links to structural impacts on inequities or really any structural interpretation or insights. You ask only about demographics, and looking at differences by sex, gender, and other demographic factors does not necessarily reflect structural effects on inequities.  It is a big jump for me that you focused on rearticulating cultural presentations in structural terms based on such a simple cross-sectional quantitative survey.

Perhaps you did ask questions that reflect structural competency and if so please describe. If what I understand is correct – that you did not use the framework in designing the study and survey, I would instead present the data and then apply some of these concepts as possible interpretations of the data. What you are getting at is potential structural impacts of the findings. Response: Thank you for the thoughtful comments. We recognize and agree that our study's cross-sectional survey-based design limits direct measurement of structural forces. We have revised the manuscript to clarify that we used structural competency as an interpretive lens rather than a design framework, to focus on identities which would be impacted the most from abortion restrictions as a structural factor.   

1. How did you ask about sex, gender, religion, race, ethnicity, etc?  Was Hispanic ethnicity asked as a separate question from race? Also, I don’t think you asked about religious importance, only religious identity.

Response: Thank you for asking these very important questions. Please note that we uploaded the actual survey for review which is publicly available here. As you may see, we tried to be inclusive when asking for gender identity and allowed a space for individuals to write if their gender was not amongst the chosen ones. We used the same approach for religion. In terms of race and ethnicity, while both were asked in one question, individuals could and did identify having more than one race and/or ethnicity. We do recognize that we should have ideally asked about ethnicity separately from race as this is an opportunity of growth for us. Regarding religious importance: You are right, that is an important distinction and for brevity we did not include that question which is a limitation to our study and has been added as such.

1. “Only 18 students voluntarily disclosed information about past abortions” – this is a very biased sentence.  It sounds like you assume more have had abortions.  The same could be true about how they reported their past pregnancies, or how many had children. I would just present the data.

Response: Thank you so much for pointing this out. We agree and have reworded that sentence and removed the word Only.

1. Why did you choose sex instead of gender? From a sociocultural and structural perspective, gender is more important. I’m assuming you asked because you were curious about differences by having the reproductive potential for pregnancy? I would make that clear. Also as I asked above how did you ask about sex and gender? Which is presented in the first paragraph of Results when you say “female”?

Response: Thank you for your comments. We asked about both gender and sex. As you pointed out, we wanted to explore differences in perceptions and beliefs among those having reproductive potential for pregnancy as the Dobbs decision would directly impact them. Please refer to previous comment on how gender and sex were asked. We would also like to point out that we did do analysis among different genders and had identical results i.e among those individuals who identified as women

1. This section is too long. You don’t need to report all the details in sentences since you have nice tables.

Response: We appreciate your suggestion and have done so.

1. I would significantly simplify your discussion.

Response: We appreciate your suggestion and have done so.

1. I am confused about the relationship between the survey results to how you are seeing structural competency.  For example, I do not think you “focus on rearticulating the cultural presentation of abortion”. You analyze the data by sex and other demographics. You found that some people with the potential to become pregnant and/or are socialized as women make different decisions and have different opinions than those without pregnancy potential and/or are socialized as man, and this may be based on protecting their reproductive autonomy and access to miscarriage and abortion care. Career decision making includes personal and other factors.  I’m also not seeing the connection with structures that support health.

Response: Thank you for this perspective.  We agree the relationship between the survey results and structural competency was unclear.  We have modified this accordingly.

1. I don’t have time to go through every paragraph, but I recommend that you focus this on the implication for medical education and the workforce.  What other studies look at career decision making.  You say “this is one of the first studies designed to collect demographic data and personal views.” Perhaps you should be more specific. Many studies about integration of abortion care in practice have included personal beliefs about abortion. What are the potential implications on health care inequities?

Response: We agree with your recommendation to be more specific and focus more on health inequities and these modifications have been made.

1. I don’t think it is a strength that you all work at IU. I do think it is a strength that you surveyed students at a large medical school in a restrictive state.

Response: Thank you for this suggestion.  We have modified accordingly.

1. I would significantly shorten all parts of the discussion. For example, you could shorten the paragraph on religion. I think you are stretching the implications – for example the last two sentences don’t really connect to the data, and I’m not sure how they relate to each other. Are you trying to say that the religious demographics of the US is proof of long lasting impacts?

Response: We have shortened the discussion throughout.  We have also simplified the discussion on religion. We have also modified the connection regarding religious demographics.  

1. your table titles should stand alone so that someone can just look at them and know what the study is about. So they should be something like: Demographics of IU medical students responding to a survey about abortion attitudes and career plans… In table 1 what does “Having children” mean?

Response: Thank you for your suggestion. We have modified the table heading per your suggestion. Having children meant currently being a biological, foster or adopted parent

# Response

**Published:** 2025-05-04

**Authors:** Zeb Saeed

**Affiliations:** Indiana University Health Inc, Indianapolis, Indiana, USA

Response to reviewer 2:

1. Thank you for the opportunity to review this manuscript, which departs from the Dobbs decision on federal restrictive abortion laws to examine its implications for maternal mortality rates and the medical field.This study aims to analyze the perceptions of third- and fourth-year medical students from one state university regarding how the state's current abortion restrictions may impact their career choices.The abstract is concise and clear. Overall, the manuscript is written in a somewhat colloquial and practical manner, making it easy to read.

Response: We thank you for reviewing our manuscript and providing feedback. We were especially intentional about making the manuscript colloquial and easy to read, so we appreciate you saying we have achieved our goal. Thank you again.

1. The authors describe their methodology, which centers on an anonymous survey conducted at Indiana University School of Medicine between December 2022 and March 2023. The survey included questions about personal beliefs on abortion, current abortion laws in Indiana, and factors influencing the choice of residency training and practice locations. However, we wonder about the process used to establish the reliability and validity of the measurement instrument. We suggest conducting a mixed-method investigation to gain a deeper understanding of the perceptions of Indiana medical students in their third and fourth years of education following the Dobbs decision. This could help assess whether the state’s current abortion restrictions impact their choices regarding residency training and where they ultimately wish to practice and live. An explanation of this process would enrich the manuscript.

Response: Thank you for your comments. We agree that in an ideal scenario, we would have either conducted a pilot survey or used a validated instrument for this study. However, to ensure the timeliness of this survey in the midst of changing laws, we decided to forego piloting the survey instead of delaying it.  At the time of the survey development in 2022, there were no validated instruments or similar surveys to emulate. Hence, we modeled several of our questions after the only publicly available survey at the time, a BestColleges' survey of undergraduate and graduate students. To identify relevant factors that may influence residency choices for students, we sought guidance from the 2021 NRMP Applicant Survey, in particular, on data on applicants pursuing OB/GYN residency. This has been added to the methods section of the manuscript. We appreciate the suggestion of having a mixed-methods study design and while this was outside of scope for this study; we would certainly consider this for future research.

1. Despite the limitations of a single-center investigation and the strategically selected population in a "more conservative state," the sample size is significant.

Response: We appreciate your comments. As you pointed out, Indiana is a more conservative state with the largest medical school in the US, hence we consider our study to be an important addition to the medical education literature

1. The tables and diagrams guide the reader through the manuscript. The survey results indicate that four-fifths of Indiana medical students disagreed with the Dobbs decision. Initially, 71.4% of students had not considered state abortion laws when selecting a medical school. However, after the Dobbs decision, 66.3% of third-year and 40.3% of fourth-year students indicated they would now consider abortion laws when choosing a residency program. Additionally, 47.5% of female students stated they would seek residency in a state where abortion is legal, and 55.3% of single students were more likely to leave Indiana to practice medicine. The discussion section includes both strengths and limitations of the research, suggesting that physicians with more liberal views on abortion are less likely to work in conservative states. The manuscript examines how socio-cultural environments can shape medical education, career choices, and practice locations. The authors warn that this trend may compromise patient care and emphasize the importance of abortion laws in shaping the healthcare workforce and influencing women's reproductive decisions and medical careers.

Response: Thank you for your kind comments and they are appreciated.

1. The manuscript aims to present a specific viewpoint and highlights associations worth reflecting on, opening a potential avenue for further investigation. This perspective is likely to capture the attention of the health professions education community and stimulate conversation by raising similar or dissenting opinions. While reading this manuscript, one might recall Freire’s statement: “There is no such thing as a neutral education process” (1). This notion prompts reflection on education as either a tool for freedom or, conversely, a means of conforming to and reinforcing the status quo.

Response: We believe we highlighted the reinforcement of the status quo in our Discussion paragraph regarding religion (paragraph 5) and also reviews the challenges facing the health professions education community on this topic.  

1. In conclusion, we believe that, despite the need for a more thorough articulation of the study's limitations, the manuscript holds inherent value and is worth reading. It may potentially inspire others to advance evidence-based perspectives in this field. Perhaps the manuscript would better fit the format of a perspective piece.

Response: We appreciate this recommendation and have expanded the limitations.

# Reviewer report: Reviewer response for version 1

**DOI:** 10.21956/mep.21961.r40972

**Published:** 2025-02-25

**Authors:** Helena Prior Filipe

**Affiliations:** 1 Egas Moniz Interdisciplinary Research Centre (Ringgold ID: 510440), Caparica, Setúbal, Portugal; 2 Ophthalmology, Hospital Centre of West Lisbon Campus Hospital of Egas Moniz, Lisboa, Lisbon, Portugal

Thank you for the opportunity to review this manuscript, which departs from the Dobbs decision on federal restrictive abortion laws to examine its implications for maternal mortality rates and the medical field.

 This study aims to analyze the perceptions of third- and fourth-year medical students from one state university regarding how the state's current abortion restrictions may impact their career choices.

 The abstract is concise and clear. Overall, the manuscript is written in a somewhat colloquial and practical manner, making it easy to read.

 The introduction provides background on the problem, which the authors tackle by conducting an online survey.

 The authors describe their methodology, which centers on an anonymous survey conducted at Indiana University School of Medicine between December 2022 and March 2023. The survey included questions about personal beliefs on abortion, current abortion laws in Indiana, and factors influencing the choice of residency training and practice locations. However, we wonder about the process used to establish the reliability and validity of the measurement instrument. We suggest conducting a mixed-method investigation to gain a deeper understanding of the perceptions of Indiana medical students in their third and fourth years of education following the Dobbs decision. This could help assess whether the state’s current abortion restrictions impact their choices regarding residency training and where they ultimately wish to practice and live. An explanation of this process would enrich the manuscript.

 Despite the limitations of a single-center investigation and the strategically selected population in a "more conservative state," the sample size is significant.

 The tables and diagrams guide the reader through the manuscript. The survey results indicate that four-fifths of Indiana medical students disagreed with the Dobbs decision. Initially, 71.4% of students had not considered state abortion laws when selecting a medical school. However, after the Dobbs decision, 66.3% of third-year and 40.3% of fourth-year students indicated they would now consider abortion laws when choosing a residency program. Additionally, 47.5% of female students stated they would seek residency in a state where abortion is legal, and 55.3% of single students were more likely to leave Indiana to practice medicine.

 The discussion section includes both strengths and limitations of the research, suggesting that physicians with more liberal views on abortion are less likely to work in conservative states. The manuscript examines how socio-cultural environments can shape medical education, career choices, and practice locations. The authors warn that this trend may compromise patient care and emphasize the importance of abortion laws in shaping the healthcare workforce and influencing women's reproductive decisions and medical careers.

 The manuscript aims to present a specific viewpoint and highlights associations worth reflecting on, opening a potential avenue for further investigation. This perspective is likely to capture the attention of the health professions education community and stimulate conversation by raising similar or dissenting opinions. While reading this manuscript, one might recall Freire’s statement: “There is no such thing as a neutral education process” (1). This notion prompts reflection on education as either a tool for freedom or, conversely, a means of conforming to and reinforcing the *status quo*.

 In conclusion, we believe that, despite the need for a more thorough articulation of the study's limitations, the manuscript holds inherent value and is worth reading. It may potentially inspire others to advance evidence-based perspectives in this field. Perhaps the manuscript would better fit the format of a perspective piece.

 Freire, P. (1988). “The Adult Literacy Process as Cultural Action for Freedom and Education and Conscientization.” In *Perspectives on Literacy*, ed. Eugene R. Kintgen, Barry M. Kroll, and Mike Rose. Carbondale, IL: Southern Illinois University Press, pp. 398-409.

Have any limitations of the research been acknowledged?

Partly

Is the work clearly and accurately presented and does it cite the current literature?

Yes

If applicable, is the statistical analysis and its interpretation appropriate?

Partly

Are all the source data underlying the results available to ensure full reproducibility?

Yes

Is the study design appropriate and is the work technically sound?

Partly

Are the conclusions drawn adequately supported by the results?

Yes

Are sufficient details of methods and analysis provided to allow replication by others?

Partly

Reviewer Expertise:

Mentorship, Faculty development with an emphasis for Social learning and Arts and Humanities, Online competency-based education and learning by Simulation, Continuing Professional Education

I confirm that I have read this submission and believe that I have an appropriate level of expertise to confirm that it is of an acceptable scientific standard, however I have significant reservations, as outlined above.

## References

[1] Freire, P. (1988). “The Adult Literacy Process as Cultural Action for Freedom and Education and Conscientization.” In Perspectives on Literacy, ed. Eugene R. Kintgen, Barry M. Kroll, and Mike Rose. Carbondale, IL: Southern Illinois University Press, pp. 398-409.

# Reviewer report: Reviewer response for version 1

**DOI:** 10.21956/mep.21961.r39184

**Published:** 2024-11-04

**Authors:** Jody Steinauer

**Affiliations:** 1 Department of Obstetrics, Gynecology and Reproductive Sciences, University of California San Francisco, San Francisco, California, USA

This paper describes a simple survey but needs significant revision.

 Most importantly, it should be much more concise, and I have concerns about the connection between the survey and your theoretical frameworks. I will try to make recommendations.

**Introduction:**

 This is too long and also does not have a concise logic between the background and the study. Let me try to make some recommendations.

 In paragraph 1, you don’t need the first two sentences… You can start with Dobbs.

 The Dobbs sentence can be shorter. I had a hard time following your logic in the next few sentences. You make a big leap from Dobbs not conferring a right to abortion to recommendations for medical education. You should clarify that. Why should we care about UME inclusion of abortion?  It is common, etc. And because ACOG and APGO recommend it. Also, I don’t think APGO went “one step further;” they simply clarified learning objectives for medical students and that they should learn it regardless of personal belief.  I don’t understand:  Conflicting priorities between law and associations are at the center of reproductive healthcare education…. I think what you are saying is that the laws causing restrictions in some states will make it hard for medical schools to ensure that medical students have access to clinical exposure to abortion care.  I don’t think the conflicting priorities are at the “center.”  There are many medical schools that – despite being in a restrictive state – have integrated education.

 In paragraph 2 you make an important point but it is also written in a way that is confusing.  You could be more straightforward.  In addition to restrictive laws limiting clinical abortion care, the laws will affect students’ access to comprehensive health care. 

 Paragraph 3 should also be shortened.  I like that you review how many medical students are training in restrictive states.  You should introduce Indiana later - in paragraph 5. It’s not clear why you are mentioning it until you are making the argument for a study in Indiana.

 Paragraph 4 should be integrated with paragraph 2. 

 You should integrate the Indiana data in paragraph 5. And I don’t think you need to make the case that the media coverage and Caitlin’s case makes us want to study Indiana. I think it’s just as important that it has a lot of medical students and it’s restrictive… The data could help us understand the experiences of medical students elsewhere.

**In summary:**

  I like that you first frame the importance of medical student education, then you talk about the fact that students are often of reproductive age and that they are patients who may need access to abortion thus it affects their health care access (combine the two paragraphs), then you talk about how many students are training in states with restrictions and link the two concepts – lack of training and also concern that they will not stay there or will leave limiting access for patients, then the link to the study.

**Methods:**

 how did you design the survey?  Did you pilot it? Did you use questions from other surveys?  I like the reflexivity paragraph but would recommend moving it down. And it should be shorter. And how did your positionality affect the study?

 I like the idea that you had a framework for analysis, but I’m confused by its application to this quantitative, cross-sectional survey.  I don’t see evidence that you used it to design the study or the survey. The questions don’t make links to structural impacts on inequities or really any structural interpretation or insights. You ask only about demographics, and looking at differences by sex, gender, and other demographic factors does not necessarily reflect structural effects on inequities.  It is a big jump for me that you focused on rearticulating cultural presentations in structural terms based on such a simple cross-sectional quantitative survey. 

 Perhaps you did ask questions that reflect structural competency and if so please describe. If what I understand is correct – that you did not use the framework in designing the study and survey, I would instead present the data and then apply some of these concepts as possible interpretations of the data. What you are getting at is potential structural impacts of the findings.

 How did you ask about sex, gender, religion, race, ethnicity, etc?  Was Hispanic ethnicity asked as a separate question from race? Also, I don’t think you asked about religious importance, only religious identity.

**Results :**

 “Only 18 students voluntarily disclosed information about past abortions” – this is a very biased sentence.  It sounds like you assume more have had abortions.  The same could be true about how they reported their past pregnancies, or how many had children. I would just present the data.

 Why did you choose sex instead of gender? From a sociocultural and structural perspective, gender is more important. I’m assuming you asked because you were curious about differences by having the reproductive potential for pregnancy? I would make that clear. Also as I asked above how did you ask about sex and gender? Which is presented in the first paragraph of Results when you say “female”?

 This section is too long. You don’t need to report all the details in sentences since you have nice tables.

**Discussion:**

 I would significantly simplify your discussion. 

 I am confused about the relationship between the survey results to how you are seeing structural competency.  For example, I do not think you “focus on rearticulating the cultural presentation of abortion”. You analyze the data by sex and other demographics. You found that some people with the potential to become pregnant and/or are socialized as women make different decisions and have different opinions than those without pregnancy potential and/or are socialized as man, and this may be based on protecting their reproductive autonomy and access to miscarriage and abortion care. Career decision making includes personal and other factors.  I’m also not seeing the connection with structures that support health.

 I don’t have time to go through every paragraph, but I recommend that you focus this on the implication for medical education and the workforce.  What other studies look at career decision making.  You say “this is one of the first studies designed to collect demographic data and personal views.” Perhaps you should be more specific. Many studies about integration of abortion care in practice have included personal beliefs about abortion. What are the potential implications on health care inequities?

 I don’t think it is a strength that you all work at IU. I do think it is a strength that you surveyed students at a large medical school in a restrictive state.

 I would significantly shorten all parts of the discussion. For example, you could shorten the paragraph on religion. I think you are stretching the implications – for example the last two sentences don’t really connect to the data, and I’m not sure how they relate to each other. Are you trying to say that the religious demographics of the US is proof of long lasting impacts? 

**Tables:**

 your table titles should stand alone so that someone can just look at them and know what the study is about. So they should be something like: Demographics of IU medical students responding to a survey about abortion attitudes and career plans… In table 1 what does “Having children” mean?

Have any limitations of the research been acknowledged?

Yes

Is the work clearly and accurately presented and does it cite the current literature?

Partly

If applicable, is the statistical analysis and its interpretation appropriate?

Partly

Are all the source data underlying the results available to ensure full reproducibility?

Partly

Is the study design appropriate and is the work technically sound?

Partly

Are the conclusions drawn adequately supported by the results?

No

Are sufficient details of methods and analysis provided to allow replication by others?

Partly

Reviewer Expertise:

Medical education, abortion care, abortion training, obstetrics and gynecology training, qualitative and quantitative research methods

I confirm that I have read this submission and believe that I have an appropriate level of expertise to state that I do not consider it to be of an acceptable scientific standard, for reasons outlined above.
